# Global exponential stability of Markovian jumping stochastic impulsive uncertain BAM neural networks with leakage, mixed time delays, and *α*-inverse Hölder activation functions

**DOI:** 10.1186/s13662-018-1553-7

**Published:** 2018-03-27

**Authors:** C. Maharajan, R. Raja, Jinde Cao, G. Ravi, G. Rajchakit

**Affiliations:** 1 Department of Mathematics, Alagappa University, Karaikudi, India; 2 Ramanujan Centre for Higher Mathematics, Alagappa University, Karaikudi, India; 3 School of Mathematics, Southeast University, Nanjing, China; 4 Dean Industry and Consultancy, Alagappa University, Karaikudi, India; 5 Department of Mathematics, Faculty of Science, Maejo University, Chiang Mai, Thailand

**Keywords:** LMIs, Markovian jumping systems, Leakage delay, Bidirectional associative memory, Discrete time neural networks, Passivity and stability analysis

## Abstract

This paper concerns the problem of enhanced results on robust finite time passivity for uncertain discrete time Markovian jumping BAM delayed neural networks with leakage delay. By implementing a proper Lyapunov–Krasovskii functional candidate, reciprocally convex combination method, and linear matrix inequality technique, we derive several sufficient conditions for varying the passivity of discrete time BAM neural networks. Further, some sufficient conditions for finite time boundedness and passivity for uncertainties are proposed by employing zero inequalities. Finally, the enhancement of the feasible region of the proposed criteria is shown via numerical examples with simulation to illustrate the applicability and usefulness of the proposed method.

## Introduction and problem statement with preliminaries

There has been a growing research interest in the field of recurrent neural networks (RNNs) largely studied by many researchers in recent years. The network architecture includes various types of neural networks such as bidirectional associative memory (BAM) neural networks, Hopfield neural networks, cellular neural networks, Cohen–Grossberg neural networks, neural and social networks which have received great attention due to their wide applications in the field of classification, signal and image processing, parallel computing, associate memories, optimization, cryptography, and so on. The bidirectional associative memory (BAM) neural network models were initially coined by Kosko, see [1, 2]. This network has an extraordinary class of RNNs which can have the ability to store bipolar vector pairs. It is composed of neurons and is arranged in two layers, one is the X-layer and the other is the Y-layer. The neurons in one layer are fully interconnected to the neurons in the other layer. The BAM neural networks are designed in such a way that, for a given external input, they can reveal only one global asymptotic or exponential stability equilibrium point. Hence, considerable efforts have been made in the study of stability analysis of neural networks, and as a credit to this, a large number of sufficient conditions have been proposed to guarantee the global asymptotic or exponential stability for the addressed neural networks.

Furthermore, the existence of time delays in the network will result in bad performance, instability or chaos. Accordingly, the time delays can be classified into two types: discrete and distributed delays. Here, we have taken both the delays into account while modeling our network system, because the length of the axon sizes is too large. So, it is noteworthy to inspect the dynamical behaviors of neural systems with both time delays, see, for instance, [3–11].

In [12], Shu et al. considered the BAM neural networks with discrete and distributed time delays. Some sufficient conditions were obtained to ensure the global asymptotic stability [12]. Also, time delays in the leakage term have great impact on the dynamic behavior of neural networks. However, so far, there have been a very few existing works on neural networks with time delay in the leakage term, see, for instance, [13–17].

Further the stability performance of state variable with leakage time delays was discussed by Lakshmanan et al. in [18]. While modeling a real nervous system, stochastic noises and parameter uncertainties are inevitable and should be taken into account. In the real nervous system, the synaptic transmission has created a noisy process brought on by apparent variation from the release of neurotransmitters and the connection weights of the neuron completely depend on undisputed resistance and capacitance values. Therefore, it is of practical significance to investigate the stochastic disruption in the stability of time-delayed neural networks with parameter uncertainties, see references cited therein [19–22]. Moreover, the hasty consequence (impulsive effect) is probable to exist in a wide variety of evolutionary processes that in turn make changes in the states abruptly at certain moments of time [23–28].

The conversion of the parameters jump will lead to a finite-state Markov process. Recently, the researchers in [29, 30] investigated the existence of Markovian jumps in BAMNNs and exploited the stochastic LKF approach, the new sufficient conditions were derived for the global exponential stability in the mean square.

The BAM-type NNs with Markovian jumping parameters and leakage terms were described by Wang et al. in [31]. In [32], a robust stability problem was studied and some delay-dependent conditions were derived for the neutral-type NNs with time-varying delays. The authors in [33–35] developed some conditions for the stability analysis of neural networks with integral inequality approach. The criteria to obtain the stability result of neural networks with time-varying delays were checked in[36–38]. It should be noted that, with all the consequences reported in the literature above, they are concerned only with Markovian jumping SNNs with Lipschitz model neuron activation functions. Up to now, very little attention has been paid to the problem of the global exponential stability of Markovian jumping SBAMNNs with non-Lipschitz type activation functions, which frequently appear in realistic neural networks. This situation motivates our present problem, i.e., *α*-inverse holder activation functions.

Our main objective of this paper is to study the delay-dependent exponential stability problem for a class of Markovian jumping uncertain BAM neural networks with mixed time delays, leakage delays, and *α*-inverse Holder activation functions under stochastic noise perturbation.

To the best of authors knowledge, so far, no result on the global exponential stability of Markovian jumping stochastic impulsive uncertain BAM neural networks with leakage, mixed time delays, and *α*-inverse Hölder activation functions has been available in the existing literature, which motivates our research to derive the following BAM neural networks:

1 $$\begin{aligned} \begin{aligned} &dx(t) = \biggl[-Cx(t-\nu_{1}) + W_{0}f\bigl(y(t)\bigr) + W_{1}g\bigl(y\bigl(t- \tau_{1}(t)\bigr)\bigr) + W_{2} \int_{t-\sigma_{1}}^{t}h\bigl(y(s)\bigr)\, ds+ I\biggr]\, dt \\ &\hphantom{dx(t) = {}}{}+ \rho_{1}\bigl(x(t-\nu_{1}),y(t),y \bigl(t-\tau _{1}(t)\bigr)\bigr),t)\, d\omega(t); \quad t>0, t\neq t_{k}, \\ &\Delta x(t_{k}) = M_{k}\bigl(x(t_{k^{-}}),x_{t_{k^{-}}} \bigr);\quad t = t_{k}, k\in\mathbb{Z}_{+}, \\ &dy(t) = \biggl[-Dy(t-\nu_{2}) + V_{0}\tilde{f}\bigl(x(t) \bigr) + V_{1}\tilde {g}\bigl(x\bigl(t-\tau_{2}(t)\bigr) \bigr) + V_{2} \int_{t-\sigma_{2}}^{t}\tilde {h}\bigl(x(s)\bigr)\, ds+ J \biggr]\, dt \\ &\hphantom{dy(t) ={}}{}+ \rho_{2}\bigl(y(t-\nu_{2}),x(t),x \bigl(t-\tau _{2}(t)\bigr)\bigr),t)\, d\tilde{\omega}(t);\quad t>0, t\neq t_{k}, \\ &\Delta y(t_{k}) = N_{k}\bigl(y(t_{k^{-}}),y_{t_{k^{-}}} \bigr); \quad t = t_{k}, k\in\mathbb{Z}_{+}, \end{aligned} \end{aligned}$$

where $x(t) = (x_{1}(t),x_{2}(t),\ldots,x_{n}(t))^{T}\in\mathbb{R}^{n}$ and $y(t) = (y_{1}(t),y_{2}(t),\ldots,y_{n}(t))^{T}\in\mathbb{R}^{n}$ denote the states at time *t*; $f(\cdot)$, $g(\cdot)$, $h(\cdot)$ and $\tilde {f}(\cdot)$, $\tilde{g}(\cdot)$, $\tilde{h}(\cdot)$ denote the neuron activation functions, $C = \operatorname{diag}\{{c_{i}}\}$, $D = \operatorname{diag}\{ {d_{j}}\}$ are positive diagonal matrices; $c_{i}>0$, $d_{j}>0$, $i,j=1,2,\ldots,n$, are the neural self inhibitions; $W_{0} = (W_{0ji})_{n\times n}$, $V_{0} = (V_{0ij})_{n\times n}$ are the connection weight matrices; $W_{1} = (W_{1ji})_{n\times n}$, $V_{1} = (V_{1ij})_{n\times n}$ are the discretely delayed connection weight matrices; and $W_{2} = (W_{2ji})_{n\times n}$, $V_{2} = (V_{2ij})_{n\times n}$ are the distributively delayed connection weight matrices; $I = (I_{1},I_{2},\ldots,I_{n})^{T}$ and $J = (J_{1},J_{2},\ldots ,J_{n})^{T}$ are the external inputs; $\tau_{1}(t)$ and $\tau_{2}(t)$ are the discrete time-varying delays which are bounded with $0<\tau_{1}(t)<\bar{\tau }_{1}$, $\dot{\tau}_{1}(t)\leq\tau_{1}<1$, and $0<\tau_{2}(t)<\bar {\tau}_{2}$, $\dot{\tau}_{2}(t)\leq\tau_{2}<1$, respectively; $\sigma_{1}$ and $\sigma_{2}$ are constant delays. The leakage delays $\nu_{1} \geq0$, $\nu_{2} \geq0$ are constants; $\rho_{1}: \mathbb {R}^{n}\times\mathbb{R}^{n}\times\mathbb{R}^{n}\times\mathbb {R}^{+} \longrightarrow\mathbb{R}^{n}$ and $\rho_{2}: \mathbb {R}^{n}\times\mathbb{R}^{n}\times\mathbb{R}^{n}\times\mathbb {R}^{+} \longrightarrow\mathbb{R}^{n}$ denote the stochastic disturbances $\omega(t) = (\omega_{1}(t),\omega_{2}(t),\ldots,\omega_{n}(t))^{T}$ and $\tilde{\omega}(t) = (\tilde{\omega}_{1}(t),\tilde{\omega }_{2}(t),\ldots,\tilde{\omega}_{n}(t))^{T}$ are *n*-dimensional Brownian motions defined on a complete probability space $(\mathbb{A}, \mathcal{F}, \{\mathcal{F}_{t}\}_{t\geq 0},\mathbb{P})$ with a filtration $\{{\mathcal{F}_{t}}\}_{t\geq0}$ satisfying the usual conditions (i.e., it is right-continuous and $\mathcal{F}_{0}$ contains all $\mathbb{P}$-null sets) and $\mathbb {E}\{{d\omega(t)}\} = \mathbb{E}\{{d\tilde{\omega}(t)}\} = 0$, $\mathbb{E}\{{d\omega^{2}(t)}\} = \mathbb{E}\{{d\tilde{\omega }^{2}(t)}\} = dt$; $M_{k}(\cdot): \mathbb{R}^{n}\times\mathbb{R}^{n} \rightarrow\mathbb{R}^{n}$, $N_{k}(\cdot): \mathbb{R}^{n}\times\mathbb {R}^{n} \rightarrow\mathbb{R}^{n}$, $k \in\mathbb{Z}_{+}$ are some continuous functions. The impulsive time $t_{k} $ satisfies $0 = t_{0} < t_{1} < \cdots< t_{k}\rightarrow\infty$, (i.e., $\lim_{k\rightarrow\infty}t_{k} = +\infty$) and $\inf_{k \in\mathbb{Z}_{+}}\{{t_{k}- t_{k-1}}\} > 0$.

The main contributions of this research work are highlighted as follows:

∗: Uncertain parameters, Markovian jumping, stochastic noises, and leakage delays are taken into account in the stability analysis of designing BAM neural networks with mixed time delays.
∗: By fabricating suitable LKF, the global exponential stability of addressed neural networks is checked via some less conserved stability conditions.
∗: For novelty, some uncertain parameters are initially handled in Lyapunov–Krasovskii functional which ensures the sufficient conditions for global exponential stability of designed neural networks.
∗: In our proposed BAM neural networks, by considering both the time delay terms, the allowable upper bounds of discrete time-varying delay is large when compared with some existing literature, see Table 1 of Example 4.1. This shows that the approach developed in this paper is brand-new and less conservative than some available results.

Suppose that the initial condition of the stochastic BAM neural networks (1) has the form $x(t) = \phi(t) $ for $t \in [-\bar{\omega},0]$ and $y(t) = \psi(t)$ for $t\in[-\bar {\tilde{\omega}},0 ]$, where $\phi(t)$ and $\psi(t)$ are continuous functions, $\bar{\omega} = \max(\bar{\tau}_{1}, \nu_{1},\sigma _{1})$ and $\bar{\tilde{\omega}} = \max(\bar{\tau_{2}},\nu _{2},\sigma_{2}) $. Throughout this section, we assume that the activation functions $f_{i}$, $\tilde{f_{j}}$, $g_{i}$, $\tilde {g_{j}}$, $h_{i}$, $\tilde{h_{j}}$; $i,j = 1,2,\ldots,n$, satisfy the following assumptions:

### Assumption 1

1. $f_{i},\tilde{f_{j}}$ are monotonic increasing continuous functions.
2. For any $\rho_{1}, \rho_{2}, \theta_{1}, \theta _{2}\in\mathbb{R}$, there exist the respective scalars $q_{\rho_{1}} > 0$, $r_{\rho_{1}} > 0$ and $q_{\rho_{2}} > 0$, $r_{\rho_{2}} > 0$ which are correlated with $\rho_{1}$, $\rho_{2}$ and $\alpha>0$, $\beta> 0$ so that
  $$\begin{aligned}& \bigl\vert f_{i}(\theta_{1}) - f_{i}( \rho_{1}) \bigr\vert \geq q_{i_{\rho _{1}}} \vert \theta_{1} - \rho_{1} \vert ^{\alpha}, \quad\forall \vert \theta_{1} - \rho_{1} \vert \leq r_{i_{\rho_{1}}}, \quad\mbox{and} \\& \bigl\vert \tilde{f}_{j}(\theta_{2}) - \tilde{f}_{j}(\rho_{2}) \bigr\vert \geq \tilde{q}_{j_{\rho_{2}}} \vert \theta_{2} - \rho_{2} \vert ^{\beta},\quad \forall \vert \theta_{2} - \rho_{2} \vert \leq\tilde {r}_{j_{\rho_{2}}}. \end{aligned}$$

### Assumption 2

$g_{i}$, $h_{i}$ and $\tilde{g}_{j}$, $\tilde {h}_{j}$ are continuous and satisfy

$$\begin{aligned}& \bigl\vert g_{i}(s_{1}) - g_{i}(s_{2}) \bigr\vert \leq e_{i} \bigl\vert f_{i}(s_{1}) - f_{i}(s_{2}) \bigr\vert ;\qquad \bigl\vert h_{i}(s_{1}) - h_{i}(s_{2}) \bigr\vert \leq k_{i} \bigl\vert f_{i}(s_{1}) - f_{i}(s_{2}) \bigr\vert ; \\& \bigl\vert \tilde{g}_{j}(\tilde{s}_{1})- \tilde{g}_{j}(\tilde{s}_{2}) \bigr\vert \leq \tilde{e}_{j} \bigl\vert \tilde{f}_{j}( \tilde{s}_{1}) - \tilde {f}_{j}(\tilde{s}_{2}) \bigr\vert ;\qquad \bigl\vert \tilde{h}_{j}(\tilde{s}_{1})- \tilde{h}_{j}(\tilde{s}_{2}) \bigr\vert \leq \tilde{k}_{j} \bigl\vert \tilde{f}_{j}( \tilde{s}_{1}) - \tilde {f}_{j}(\tilde{s}_{2}) \bigr\vert , \end{aligned}$$

$\forall s_{1}, s_{2}, \tilde{s}_{1}, \tilde{s}_{2} \in\mathbb{R}$, $s_{1}\neq s_{2}$ and $\tilde{s}_{1}\neq\tilde{s}_{2}$, $i,j = 1,2,3,\ldots,n$. Denote $E = \operatorname{diag}\{{e_{i}}\}$, $K = \operatorname{diag}\{ {k_{i}}\}$ and $\widetilde {E} = \operatorname{diag} \{{\tilde{e}_{j}}\}$, $\widetilde{K} = \operatorname{diag}\{{\tilde {k}_{j}}\}$ respectively.

### Remark 1.1

In [39], the function $f_{i}$ used in Assumption 1 is said to be an *α*-inverse Holder activation function which is a non-Lipschitz function. This activation function plays an important role in the stability issues of neural networks, and there exists a great number of results in the engineering mathematics, for example, $f(\theta) = \operatorname{arc} \tan\theta$ and $f(\theta)=\theta^{3} + \theta$ are 1-inverse Holder functions, $f(\theta)=\theta^{3}$ is 3-inverse Holder function.

### Remark 1.2

From Assumption 2, we can get that $e_{i}$, $\tilde{e}_{j}$ and $k_{i}$, $\tilde{k}_{j}$ are positive scalars. So *E*, *Ẽ* and *K*, *K̃* are both positive definite diagonal matrices. The relations among the different activation functions $f_{i}$, $\tilde{f}_{j}$ (which are *α*-inverse Holder activation functions) $g_{i}$, $\tilde{g}_{j}$ and $h_{i}$, $\tilde {h}_{j}$ are implicitly established in Theorem 3.2. Such relations, however, have not been provided by any of the authors in the reported literature.

In order to guarantee the global exponential stability of system (1), we assume that the system tends to its equilibrium point and the stochastic noise contribution vanishes, i.e.,

### Assumption 3

$\rho(x^{*},y^{*},y^{*},t) = 0$; $i,j = 1,2,\ldots,n$.

For such deterministic BAM neural networks, we have the following system of equations:

2 $$\begin{aligned} &dx(t) = \biggl[-Cx(t-\nu_{1})+W_{0}f \bigl(y(t)\bigr)+W_{1}\bigl(g\bigl(y\bigl(t-\tau _{1}(t) \bigr)\bigr)\bigr) \\ &\hphantom{dx(t) ={}}{}+W_{2} \int_{t-\sigma_{1}}^{t}h\bigl(y(s)\bigr)\, ds+I\biggr]\, dt, \quad t>0, t \neq t_{k}, \\ &\Delta x(t_{k}) = M_{k}\bigl(x(t_{k^{-}}),x_{t_{k^{-}}} \bigr); \quad t = t_{k}, k\in\mathbb{Z}_{+}, \\ &dy(t) = \biggl[-Dy(t-\nu_{2})+V_{0}\tilde{f}\bigl(x(t) \bigr)+V_{1}\tilde {g}\bigl(x\bigl(t-\tau_{2}(t)\bigr)\bigr) \\ &\hphantom{dy(t) ={}}{}+V_{2} \int_{t-\sigma_{2}}^{t}\tilde {h}\bigl(x(s)\bigr)\, ds+J\biggr] \,dt,\quad t>0, t \neq t_{k}, \\ &\Delta y(t_{k}) = N_{k}\bigl(y(t_{k^{-}}),y_{t_{k^{-}}} \bigr);\quad t = t_{k}, k\in\mathbb{Z}_{+}. \end{aligned}$$

Thus system (1) admits one equilibrium point $(x^{*},y^{*}) = (x_{1}^{*},x_{2}^{*},\ldots,x_{n}^{*},y_{1}^{*},y_{2}^{*},\ldots,y_{n}^{*})^{T}$ under Assumption 3. In this regard, let $u(t) = x(t)-x^{*}$ and $v(t) = y(t)-y^{*}$, then system (1) can be rewritten in the following form:

3 $$\begin{aligned} \begin{aligned} &du(t) = \biggl[-Cu(t-\nu_{1})+W_{0} \bar{f}\bigl(v(t)\bigr)+W_{1}\bigl(\bar{g}\bigl(v\bigl(t-\tau _{1}(t)\bigr)\bigr)\bigr)+W_{2} \int_{t-\sigma_{1}}^{t}\bar{h}\bigl(v(s)\bigr)\, ds\biggr]\, dt \\ &\hphantom{du(t) ={}}{}+\bar{\rho_{1}}\bigl(u(t-\nu_{1}),v(t),v \bigl(t-\tau _{1}(t)\bigr),t\bigr)\,d\bar {\omega}(t),\quad t>0, t \neq t_{k}, \\ &\Delta u(t_{k}) = \bar{M}_{k}\bigl(u\bigl(t_{k}^{-} \bigr),u_{t_{k}^{-}}\bigr),\quad t=t_{k}, k\in\mathbb{Z}_{+}, \\ &dv(t) = \biggl[-Dv(t-\nu_{2})+V_{0}\bar{\tilde{f}} \bigl(u(t)\bigr)+V_{1}\bar {\tilde{g}}\bigl(u\bigl(t- \tau_{2}(t)\bigr)\bigr)+V_{2} \int_{t-\sigma_{2}}^{t}\bar {\tilde{h}}\bigl(u(s)\bigr)\, ds \biggr]\, dt \\ &\hphantom{dv(t) = {}}{}+\bar{\rho_{2}}\bigl(v(t-\nu_{2}),u(t),u \bigl(t-\tau _{2}(t)\bigr),t\bigr)\, d\bar {\tilde{\omega}}(t),\quad t>0, t \neq t_{k}, \\ &\Delta v(t_{k}) = \bar{N}_{k}\bigl(v\bigl(t_{k}^{-} \bigr),v_{t_{k}^{-}}\bigr),\quad t=t_{k}, k\in\mathbb{Z}_{+}, \end{aligned} \end{aligned}$$

where

$$\begin{aligned}& u(t) = \bigl(u_{1}(t),u_{2}(t),\ldots,u_{n}(t) \bigr)^{T} , \\& v(t) = \bigl(v_{1}(t),v_{2}(t),\ldots,v_{n}(t) \bigr)^{T} , \\& u(t-\nu_{1}) = \bigl(u_{1}(t-\nu_{1}),u_{2}(t- \nu_{1}),\ldots,u_{n}(t-\nu _{1}) \bigr)^{T} , \\& v(t-\nu_{2}) = \bigl(v_{1}(t-\nu_{2}),v_{2}(t- \nu_{2}),\ldots,v_{n}(t-\nu _{2}) \bigr)^{T} , \\& \bar{f}\bigl(v(t)\bigr) = \bigl(\bar{f}_{1}\bigl(v(t)\bigr), \bar{f}_{2}\bigl(v(t)\bigr),\ldots, \bar {f}_{n}\bigl(v(t) \bigr)\bigr)^{T} , \\& \bar{\tilde{f}}\bigl(u(t)\bigr) = \bigl(\bar{\tilde{f}}_{1}\bigl(u(t) \bigr),\bar{\tilde {f}}_{2}\bigl(u(t)\bigr),\ldots,\bar{ \tilde{f}}_{n}\bigl(u(t)\bigr)\bigr)^{T} , \\& \bar{g}\bigl(v\bigl(t-\tau_{1}(t)\bigr)\bigr) = \bigl( \bar{g}_{1}\bigl(v\bigl(t-\tau_{1}(t)\bigr)\bigr),\bar {g}_{2}\bigl(v\bigl(t-\tau_{1}(t)\bigr)\bigr),\ldots, \bar{g}_{n}\bigl(v\bigl(t-\tau_{n}(t)\bigr)\bigr) \bigr)^{T} , \\& \bar{\tilde{g}}\bigl(u\bigl(t-\tau_{2}(t)\bigr)\bigr)=\bigl(\bar{ \tilde{g}}_{1}\bigl(u\bigl(t-\tau _{2}(t)\bigr)\bigr),\bar{ \tilde{g}}_{2}\bigl(u\bigl(t-\tau_{2}(t)\bigr)\bigr),\ldots, \bar{\tilde {g}}_{n}\bigl(u\bigl(t-\tau_{n}(t)\bigr)\bigr) \bigr)^{T} , \\& \bar{h}\bigl(v(t)\bigr) = \bigl(\bar{h}_{1}\bigl(v(t)\bigr), \bar{h}_{2}\bigl(v(t)\bigr),\ldots,\bar {h}_{n}\bigl(v(t) \bigr)\bigr)^{T} , \\& \bar{\tilde{h}}\bigl(u(t)\bigr) = \bigl(\bar{\tilde{h}}_{1}\bigl(u(t) \bigr),\bar{\tilde {h}}_{2}\bigl(u(t)\bigr),\ldots,\bar{ \tilde{h}}_{n}\bigl(u(t)\bigr)\bigr)^{T} , \\& \bar{f}_{i}\bigl(v(t)\bigr) = f_{i}\bigl(v(t)+y^{*} \bigr)-f_{i}\bigl(y^{*}\bigr) , \\& \bar{\tilde{f}}_{j}\bigl(u(t)\bigr) = \tilde{f}_{j} \bigl(u(t)+x^{*}\bigr)-\tilde {f}_{j}\bigl(x^{*} \bigr) , \\& \bar{g}_{i}\bigl(v\bigl(t-\tau_{1}(t)\bigr)\bigr)= g_{i}\bigl(v\bigl(t-\tau _{1}(t)\bigr)+y^{*} \bigr)-g_{i}\bigl(y^{*}\bigr) , \\& \bar{\tilde{g}}_{j}\bigl(u\bigl(t-\tau_{2}(t)\bigr)\bigr) = \tilde{g}_{j}\bigl(u\bigl(t-\tau _{2}(t) \bigr)+x^{*}\bigr)-\tilde{g}_{j}\bigl(x^{*}\bigr) , \\& \bar{h}_{i}\bigl(v(t)\bigr)= h_{i}\bigl(v(t)+y^{*} \bigr)-h_{i}\bigl(y^{*}\bigr) , \\& \bar{\tilde{h}}_{j}\bigl(u(t)\bigr) =\tilde{h}_{i} \bigl(u(t)+x^{*}\bigr)-\tilde {h}_{i}\bigl(x^{*} \bigr) , \\& \bar{\rho}_{1}\bigl(u(t-\nu_{1}),v(t),v(t- \tau_{1}),t\bigr) \\& \quad= \rho_{1}\bigl(u(t-\nu_{1})+x^{*},v(t)+y^{*},v \bigl(t-\tau _{1}(t)\bigr)+y^{*},t\bigr)-\rho _{1}\bigl(x^{*},y^{*},y^{*},t\bigr) , \\& \bar{\rho}_{2}\bigl(v(t-\nu_{2}),u(t),u(t- \tau_{2}),t\bigr) \\& \quad= \rho_{2}\bigl(v(t-\nu_{2})+y^{*},u(t)+x^{*},u \bigl(t-\tau _{2}(t)\bigr)+x^{*},t\bigr)-\rho _{2} \bigl(y^{*},x^{*},x^{*},t\bigr) , \\& \bar{\rho}_{1}\bigl(u(t-\nu_{1}),v(t),v(t- \tau_{1}),t\bigr) \\& \quad= \bigl(\bar{\rho}_{11}\bigl(u(t-\nu_{1}),v(t),v \bigl(t-\tau_{1}(t)\bigr),t\bigr),\ldots, \bar{\rho}_{1n} \bigl(u(t-\nu_{1}),v(t),v\bigl(t-\tau_{1}(t)\bigr),t\bigr) \bigr)^{T} , \\& \bar{\rho}_{2}\bigl(v(t-\nu_{2}),u(t),u(t- \tau_{2}),t\bigr) \\& \quad= \bigl(\bar{\rho }_{21}\bigl(v(t-\nu_{2}),u(t) , u \bigl(t-\tau_{2}(t)\bigr),t\bigr),\ldots, \bar{\rho}_{2n} \bigl(v(t-\nu _{2}),u(t),u\bigl(t-\tau_{2}(t)\bigr),t\bigr) \bigr)^{T} . \end{aligned}$$

Apparently, $\bar{f}_{i}(s)$, $\bar{\tilde{f}}_{j}(s)$ is also an *α*-inverse Holder function, and $\bar{f}_{i}(0) = \bar {g}_{i}(0) = \bar{h}_{i}(0) = \bar{\tilde{f}}_{j}(0) = \bar{\tilde{g}}_{j}(0) = \bar{\tilde{h}}_{j}(0) = 0$, $i,j=1,2,\ldots,n$.

Let $\{r(t), t \geq0 \}$ be a right-continuous Markov chain in a complete probability space $(\Omega,\mathcal{F},\{\mathcal{F}_{t}\} _{t\geq0},\mathbb{P})$ and take values in a finite state space $\mathfrak{M} = \{1,2,\ldots,N\}$ with generator $\Gamma= (\gamma_{ij})_{N\times N}$ given by

$$ P\bigl\{ r(t+\Delta t) = j|r(t) = i\bigr\} = \textstyle\begin{cases} \gamma_{ij} \Delta t +O(\Delta t), & \mbox{if } i \neq j, \\ 1+\gamma_{ii} \Delta t +O(\Delta t), & \mbox{if } i=j, \end{cases} $$

where $\Delta t > 0$ and $\lim_{\Delta t\rightarrow0}(\frac{O(\Delta t)}{\Delta t})=0$. Here $\gamma_{ij} \geq0$ is the transition probability rate from *i* to *j* if $i \neq j$, while $\gamma_{ii} = -\sum_{j=1}^{N}\gamma_{ij}$.

In this paper, we consider the following BAM neural networks with stochastic noise disturbance, leakage, mixed time delays, and Markovian jump parameters, which is actually a modification of system (3):

4 $$\begin{aligned} \begin{aligned} &du(t) = \biggl[-C\bigl(r(t)\bigr)u(t- \nu_{1})+W_{0}\bigl(r(t)\bigr)\bar {f}\bigl(v(t) \bigr) \\ &\hphantom{du(t) ={}}{}+W_{1}\bigl(r(t)\bigr) \bigl(\bar{g}\bigl(v\bigl(t- \tau_{1}(t)\bigr)\bigr)\bigr) +W_{2}\bigl(r(t)\bigr) \int_{t-\sigma_{1}}^{t}\bar{h}\bigl(v(s)\bigr)\,ds\biggr]\, dt \\ &\hphantom{du(t) ={}}{}+ \bar{\rho _{1}}\bigl(u(t-\nu_{1}), v(t),v\bigl(t- \tau_{1}(t)\bigr), t,r(t)\bigr)\, d\bar{\omega}(t), \quad t>0, t \neq t_{k}, \\ &\Delta u(t_{k}) = \bar{M}_{k}\bigl(r(t)\bigr) \bigl(u \bigl(t_{k}^{-}\bigr),u_{t_{k}^{-}}\bigr),\quad t=t_{k}, k\in\mathbb{Z}_{+}, \\ &dv(t) = \biggl[-D\bigl(\tilde{r}(t)\bigr)v(t-\nu_{2})+V_{0} \bigl(\tilde{r}(t)\bigr)\bar {\tilde{f}}\bigl(u(t)\bigr) \\ &\hphantom{dv(t) ={}}{}+V_{1}\bigl( \tilde{r}(t)\bigr) \bar{\tilde{g}}\bigl(u\bigl(t-\tau_{2}(t)\bigr) \bigr)+V_{2}\bigl(\tilde{r}(t)\bigr) \int_{t-\sigma_{2}}^{t}\bar{\tilde {h}}\bigl(u(s)\bigr)\,ds \biggr]\,dt \\ &\hphantom{dv(t) ={}}{}+\bar {\rho_{2}}\bigl(v(t-\nu_{2}), u(t),u\bigl(t- \tau_{2}(t)\bigr), t,\tilde{r}(t)\bigr)\, d\bar{\tilde{\omega}}(t), \quad t>0, t \neq t_{k}, \\ &\Delta v(t_{k}) = \bar{N}_{k}\bigl(\tilde {r}(t)\bigr) \bigl(v(t_{k^{-}}),v_{t_{k^{-}}}\bigr), \quad t = t_{k}, k\in \mathbb {Z}_{+}, \end{aligned} \end{aligned}$$

where $u(t-\nu_{1})$, $\tau_{1}(t)$, $\tau_{2}(t)$, $v(t)$, $u(t)$, $v(t-\nu_{2})$, $\bar{f}(v(t))$, $\bar{\tilde{f}}(u(t))$, $\bar {g}(v(t-\tau_{1}(t)))$, $\bar{\tilde{g}}(u(t-\tau_{2}(t)))$, $\bar {h}(v(t))$, $\bar{\tilde{h}}(u(t))$ have the same meanings as those in (3), $\bar{\rho_{1}}(u(t-\nu_{1}), v(t), v(t-\tau _{1}(t)), t,r(t))$ and $\bar{\rho_{2}}(v(t-\nu _{2}),u(t),u(t-\tau_{2}(t)),t,\tilde{r}(t))$ are noise intensity function vectors, and for a fixed system mode, $C(r(t))$, $D(r(t)) $, $W_{0}(r(t))$, $V_{0}(\tilde{r}(t))$, $W_{1}(r(t))$, $V_{1}(\tilde{r}(t))$, $W_{2}(r(t))$, $V_{2}(\tilde{r}(t))$, $\bar {M}_{k}(r(t))$, and $\bar{\tilde{N}}_{k}(\tilde{r}(t))$ are known constant matrices with appropriate dimensions.

For our convenience, each possible value of $r(t)$ and $\tilde {r}(t)$ is denoted by *i* and *j* respectively; $i, j \in\mathfrak {M}$ in the sequel. Then we have $C_{i} = C(r(t))$, $D_{j} = D(\tilde{r}(t))$, $W_{0i}=W_{0}(r(t))$, $V_{0j}= V_{0}(\tilde{r}(t))$, $W_{1i}=W_{1}(r(t))$, $V_{1j}= V_{1}(\tilde{r}(t))$, $W_{2i}=W_{2}(r(t))$, $V_{2j}= V_{2}(\tilde{r}(t))$, $\bar{M}_{ki}=\bar{M}_{k}(r(t))$, $\bar {\tilde{N}}_{kj}=\bar{\tilde{N}}_{k}(\tilde{r}(t))$, where $C_{i}$, $D_{j}$, $W_{0i}$, $V_{0j}$, $W_{1i}$, $V_{1j}$, $W_{2i}$, $V_{2j}$, $\bar{M}_{ki}$, $\bar{\tilde{N}}_{kj}$ for any $i, j\in \mathfrak{M}$.

Assume that $\bar{\rho_{1}} : \mathbb{R}^{n} \times \mathbb{R}^{n}\times\mathbb{R}^{n}\times\mathbb {R}^{+}\times\mathfrak{M}\rightarrow\mathbb{R}^{n}$ and $\bar{\rho_{2}} : \mathbb{R}^{n}\times\mathbb{R}^{n}\times\mathbb {R}^{n}\times\mathbb{R}^{+}\times \mathfrak{M}\rightarrow\mathbb{R}^{n}$ are locally Lipschitz continuous and satisfy the following assumption.

### Assumption 4

$$\begin{aligned}& \operatorname{trace} \bigl[\bar{\rho_{1}}^{T}(u_{1},v_{1},v_{2},t,i) \bar{\rho _{1}}(u_{1},v_{1},v_{2},t,i) \bigr] \leq u_{1}^{T}R_{1i}u_{1}+v_{1}^{T}R_{2i}v_{1}+v_{2}^{T}R_{3i}v_{2}; \\& \operatorname{trace} \bigl[\bar{\rho_{2}}^{T}(v_{1},u_{1},u_{2},t,j) \bar{\rho _{2}}(v_{1},u_{1},u_{2},t,j) \bigr] \leq v_{1}^{T}\widetilde {R}_{1j}v_{1}+u_{1}^{T} \widetilde{R}_{2j}u_{1}+u_{2}^{T} \widetilde {R}_{3j}u_{2}; \end{aligned}$$

for all $u_{1}, u_{2}, v_{1}, v_{2}\in\mathbb{R}^{n}$ and $r(t)=i$, $\tilde{r}(t)=j$, $i, j\in\mathfrak {M}$,where $R_{1i}$, $\widetilde{R}_{1j}$, $R_{2i}$, $\widetilde {R}_{2j}$, $R_{3i}$, and $\widetilde{R}_{3j}$ are known positive definite matrices with appropriate dimensions.

Consider a general stochastic system $dx(t)=f(x(t), t, r(t))\, dt + g(x(t), t, r(t))\, d\omega(t)$, $t \geq0$ with the initial value $x(0) = x_{0} \in\mathbb{R}^{n}$, where $f: \mathbb {R}^{n}\times\mathbb{R}^{+}\times\mathfrak{M} \rightarrow \mathbb{R}^{n}$ and $r(t)$ is the Markov chain. Let $C^{2,1}(\mathbb {R}^{n} \times\mathbb{R}^{+} \times\mathfrak{M}; \mathbb {R}^{+})$ denote a family of all nonnegative functions *V* on $\mathbb {R}^{n}\times\mathbb{R}^{+}\times\mathfrak{M}$ which are twice continuously differentiable in *x* and once differentiable in *t*. For any $V \in C^{2,1}(\mathbb{R}^{n}\times\mathbb{R}^{+}\times\mathfrak {M}; \mathbb{R}^{+})$, define $\mathcal{L}V:\mathbb{R}^{n}\times \mathbb{R}^{+} \times\mathfrak{M} \rightarrow\mathbb{R}$ by

$$\begin{aligned} \mathcal{L}V\bigl(x(t),t,i\bigr) =& V_{t}\bigl(x(t),t,i\bigr) + V_{x}\bigl(x(t),t,i\bigr)f\bigl(x(t),t,i\bigr) \\ &{} +\frac{1}{2} \operatorname{trace} \bigl(g^{T} \bigl(x(t),t,i\bigr) V_{xx}\bigl(x(t),t,i\bigr) g\bigl(x(t),t,i\bigr) \bigr) \\ &{}+\sum_{j=1}^{N} \gamma_{ij}V \bigl(x(t),t,j\bigr), \end{aligned}$$

where

$$\begin{aligned}& V_{t}\bigl(x(t),t,i\bigr) = \frac{\partial V(x(t),t,i)}{\partial t}, \\& V_{x}\bigl(x(t),t,i\bigr) = \biggl(\frac{\partial V(x(t),t,i)}{\partial x_{1}}, \frac{\partial V(x(t),t,i)}{\partial x_{2}},\ldots,\frac{\partial V(x(t),t,i)}{\partial x_{n}} \biggr) , \\& V_{xx}\bigl(x(t),t,i\bigr) = \frac{\partial^{2} V(x(t),t,i)}{\partial x_{j}\, \partial x_{k}}. \end{aligned}$$

By generalized Ito’s formula, one can see that

$$ \mathbb{E}V\bigl(x(t),y(t),t,r(t)\bigr) = \mathbb{E}V\bigl(x(0),y(0),0,r(0) \bigr) + \mathbb{E} \int_{0}^{t} \mathcal{L}V\bigl(x(s),y(s),s,r(s) \bigr)\,ds. $$

Let $u(t;\xi)$ and $v(t;\tilde{\xi})$ denote the state trajectory from the initial data $u(\theta)=\xi(\theta)$ on $-\bar{\omega}\leq\theta\leq0$ in $L_{\mathcal {F}_{0}}^{2}([-\bar{\omega},0];\mathbb{R}^{n})$ and $v(\theta)=\tilde{\xi}(\theta)$ on $-\bar{\tilde{\omega}}\leq\theta \leq0$ in $L_{\mathcal{F}_{0}}^{2}([-\bar{\tilde{\omega }},0];\mathbb{R}^{n})$. Clearly, system (4) admits a trivial solution $u(t,0)\equiv0$ and $v(t,0)\equiv0$ corresponding to the initial data $\xi=0$ and $\tilde{\xi}=0$, respectively. For simplicity, we write $u(t;\xi)=u(t)$ and $v(t,\tilde{\xi})=v(t)$.

### Definition 1.3

The equilibrium point of neural networks (4) is said to be globally exponentially stable in the mean square if, for any $\xi\in L_{\mathcal{F}_{0}}^{2}([-\bar{\omega },0];\mathbb{R}^{n})$, $\tilde{\xi} \in L_{\mathcal {F}_{0}}^{2}([-\bar{\tilde{\omega}},0];\mathbb{R}^{n})$, there exist positive constants *η*, $\mathcal{T}$, $\Pi_{\xi}$, and $\Theta_{\tilde{\xi}}$ correlated with *ξ* and *ξ̃* such that, when $t > \mathcal{T}$, the following inequality holds:

$$\mathbb{E}\bigl\{ \bigl\Vert u(t;\xi) \bigr\Vert ^{2}\bigr\} + \mathbb{E}\bigl\{ \bigl\Vert v(t;\tilde{\xi }) \bigr\Vert ^{2}\bigr\} \leq (\Pi_{\xi}+ \Theta_{\tilde{\xi}}) e^{-\eta t} . $$

### Definition 1.4

We introduce the stochastic Lyapunov–Krasovskii functional $V \in C^{2,1}(\mathbb{R}^{+}\times\mathbb{R}^{n} \times\mathbb {R}^{n} \times\mathfrak{M};\mathbb{R}^{+})$ of system (4), the weak infinitesimal generator of random process $\mathcal{L}V$ from $\mathbb{R}^{+}\times\mathbb{R}^{n} \times\mathbb{R}^{n} \times \mathfrak{M}$ to $\mathbb{R}^{+}$ defined by

$$\begin{aligned} \mathcal{L}V\bigl(t,u(t),v(t),i\bigr) =& \lim_{\Delta t\rightarrow0^{+}} \frac {1}{\Delta t}\bigl[\mathbb{E}\bigl\{ V\bigl((t+\Delta t),u(t+\Delta t),v(t+ \Delta t), r(t+\Delta t)\bigr)| \\ & u(t),v(t),r(t) = i\bigr\} - V\bigl(t,u(t),v(t),r(t) = i\bigr)\bigr]. \end{aligned}$$

### Lemma 1.5

([39])

*If* $f_{i}$ *is an* *α*-*inverse Holder function*, *then for any* $\rho_{0} \in\mathbb{R}$, *one has*

$$ \int_{\rho_{0}}^{+\infty}\bigl[f_{i}(\theta) - f_{i}(\rho_{0})\bigr]\,d\theta = \int_{\rho_{0}}^{-\infty}\bigl[f_{i}(\theta) - f_{i}(\rho _{0})\bigr]\,d\theta= +\infty. $$

### Lemma 1.6

([39])

*If* $f_{i}$ *is an* *α*-*inverse Holder function and* $f_{i}(0) = 0$, *then there exist constants* $q_{i_{0}} > 0$ *and* $r_{i_{0}}\geq0$ *such that* $|f_{i}(\theta)| \geq q_{i_{0}}|\theta|^{\alpha}$, $\forall|\theta| \leq r_{i_{0}}$. *Moreover*, $|f_{i}(\theta)| \geq q_{i_{0}} r_{i_{0}}^{\alpha}$, $\forall|\theta| \geq r_{i_{0}}$.

### Lemma 1.7

([21])

*For any real matrix* $M >0$, *scalars* *a* *and* *b* *with* $0 \leq a < b$, *vector function* $x(\alpha)$ *such that the following integrals are well defined*, *we have*

$$ \int_{-a}^{-b} \int_{t+\beta}^{t} x(\alpha)^{T} M x(\alpha) \,d\alpha\,d\beta\leq (b-a) \int_{t - b}^{t} x(\alpha)^{T} M x(\alpha) \,d\alpha. $$

### Lemma 1.8

([39])

*Let* $x,y \in\mathbb{R}^{n}$, *and* *G* *is a positive definite matrix*, *then*

$$ 2x^{T}y \leq x^{T} G x + y^{T} G^{-1} y. $$

### Lemma 1.9

([21])

*Given constant symmetric matrices* $\Upsilon_{1}$, $\Upsilon_{2}$, *and* $\Upsilon_{3}$ *with appropriate dimensions*, *where* $\Upsilon _{1}^{T}=\Upsilon_{1}$ *and* $\Upsilon_{2}^{T}=\Upsilon_{2} > 0$, $\Upsilon_{1}+\Upsilon_{3}^{T} \Upsilon_{2}^{-1} \Upsilon_{3} < 0$ *if and only if*

$$ \begin{bmatrix} \Upsilon_{1}& \Upsilon_{3}^{T}\\ \Upsilon_{3}& -\Upsilon_{2} \end{bmatrix} < 0 \quad [\textit{or}]\quad \begin{bmatrix} \Upsilon_{1}& \Upsilon_{3}\\ \Upsilon_{3}^{T}& -\Upsilon_{2} \end{bmatrix} < 0. $$

### Lemma 1.10

([21])

*For any constant matrix* $\Omega\in\mathbb{R}^{n \times n}$, $\Omega = \Omega^{T} > 0$, *scalar* $\gamma> 0$, *vector function* $\omega:[0, \gamma] \rightarrow\mathbb{R}^{n}$, *such that the integrations concerned are well defined*, *then*

$$ \frac{1}{\gamma} \biggl( \int_{0}^{\gamma} \omega(s) \,ds \biggr)^{T} \Omega \biggl( \int_{0}^{\gamma} \omega(s)\,ds \biggr) \leq \int _{0}^{\gamma} \omega^{T}(s) \Omega \omega(s) \,ds. $$

### Lemma 1.11

([33])

*For given matrices* *D*, *E*, *and* *F* *with* $F^{T}F \leq I$ *and scalar* $\epsilon> 0$, *the following inequality holds*:

$$ DFE + E^{T} F^{T} D^{T} \leq\epsilon DD^{T} +\epsilon^{-1} EE^{T}. $$

### Remark 1.12

Lakshmanan et al. in [18] analyzed the impact of time-delayed BAM neural networks for ensuring the stability performance when the leakage delay occurred. In [12], the authors discussed the stability behavior in the sense of asymptotic for BAM neural networks with mixed time delays and uncertain parameters. Moreover, the comparisons for maximum allowable upper bounds of discrete time-varying delays have been listed. Lou and Cui in [29] conversed the exponential stability conditions for time-delayed BAM NNs while Markovian jump parameters arose. Further, the stochastic effects on neural networks and stability criteria were conversed via exponential sense by Huang and Li in [40] by the aid of Lyapunov–Krasovskii functionals. In all the above mentioned references, the stability problem for BAM neural networks was considered only with leakage delays or mixed time delays, or stochastic effects, or Markovian jump parameters, or parameter uncertainties, but all the above factors have not been taken into one account and no one investigated exponential stability via delays at a time. Considering the above facts is very challenging and advanced in this research work.

## Global exponential stability for deterministic systems

### Theorem 2.1

*Under Assumptions* 1 *and* 2, *the neural network system* (4) *is globally exponentially stable in the mean square if*, *for given* $\eta_{i},\widetilde{\eta}_{j}>0$ ($i,j\in\mathfrak {M}$), *there exist positive definite matrices* *S*, *T*, *S̃*, *T̃*, $R_{2}$, $\widetilde{R}_{2}$, $N_{1}$, $N_{2}$, $N_{3}$, $N_{4}$, $N_{5}$, $N_{6}$ *and* $H_{i}$, $\widetilde{H}_{j}$ ($i,j\in\mathfrak{M}$), *positive definite diagonal matrices* *P*, *Q*, *and positive scalars* $\lambda_{i}$ *and* $\mu_{j}$ ($i,j\in\mathfrak {M}$) *such that the following LMIs are satisfied*:

5 $$\begin{aligned}& H_{i} < \lambda_{i}I, \end{aligned}$$

6 $$\begin{aligned}& \widetilde{H}_{j} < \mu_{j}I, \end{aligned}$$

7 $$\begin{aligned}& \bar{M}_{k}^{T}H_{i}\bar{M}_{k} - H_{j} \leq0, \end{aligned}$$

8 $$\begin{aligned}& \bar{N}_{k}^{T}\widetilde{H}_{i} \bar{N}_{k} - \widetilde{H}_{j} \leq0\quad \bigl[ \textit{here } r(t_{k})=i \textit{ and } \tilde {r}(t_{k})=j \bigr], \end{aligned}$$

9 $$\begin{aligned}& \Xi_{i}= \left [ \textstyle\begin{array}{@{}c@{\quad}c@{\quad}c@{\quad}c@{\quad}c@{\quad}c@{\quad }c@{\quad}c@{\quad}c@{\quad}c@{\quad}c@{\quad}c@{\quad}c@{\quad}c@{}} \Xi_{11}& \Xi_{12}& \Xi_{13}& 0& 0& 0& 0& 0& \Xi_{19}& 0& 0& 0& 0& 0\\ \ast& \Xi_{22}& 0& 0& \Xi_{25}& \Xi_{26}& \Xi_{27}& 0& \Xi_{29}& 0& 0& 0& 0& 0\\ \ast& \ast& \Xi_{33}& 0& \Xi_{35}& \Xi_{36}& \Xi_{37}& 0& 0& 0& 0& 0& 0& 0\\ \ast& \ast& \ast& \Xi_{44}& 0& 0& 0& 0& 0& 0& 0& 0& 0& 0\\ \ast& \ast& \ast& \ast& \Xi_{55}& 0& 0& 0& \Xi_{59}& 0& 0& 0& 0& 0\\ \ast& \ast& \ast& \ast& \ast& \Xi_{66}& 0& 0& \Xi_{69}& 0& 0& 0& 0& 0\\ \ast& \ast& \ast& \ast& \ast& \ast& \Xi_{77}& 0& \Xi_{79}& 0& 0& 0& 0& 0\\ \ast& \ast& \ast& \ast& \ast& \ast& \ast& \Xi_{88}& 0& 0& 0& 0& 0& 0\\ \ast& \ast& \ast& \ast& \ast& \ast& \ast& \ast& \Xi_{99}& 0& 0& 0& 0& 0\\ \ast& \ast& \ast& \ast& \ast& \ast& \ast& \ast& \ast& \Xi _{1010}& 0& 0& 0& 0\\ \ast& \ast& \ast& \ast& \ast& \ast& \ast& \ast& \ast& \ast& \Xi_{1111}& 0& 0& 0\\ \ast& \ast& \ast& \ast& \ast& \ast& \ast& \ast& \ast& \ast& \ast& \Xi_{1212}& 0& 0\\ \ast& \ast& \ast& \ast& \ast& \ast& \ast& \ast& \ast& \ast& \ast& \ast& \Xi_{1313}& 0\\ \ast& \ast& \ast& \ast& \ast& \ast& \ast& \ast& \ast& \ast& \ast& \ast& \ast& \Xi_{1414} \end{array}\displaystyle \right ] \\& \hphantom{\Xi_{i}} < 0, \end{aligned}$$

10 $$\begin{aligned}& \Omega_{j}= \left [ \textstyle\begin{array}{@{}c@{\quad}c@{\quad}c@{\quad}c@{\quad}c@{\quad}c@{\quad }c@{\quad}c@{\quad}c@{\quad}c@{\quad}c@{\quad}c@{\quad}c@{\quad}c@{}} \Omega_{11}& \Omega_{12}& \Omega_{13}& 0& 0& 0& 0& 0& \Omega_{19}& 0& 0& 0& 0& 0\\ \ast& \Omega_{22}& 0& 0& \Omega_{25}& \Omega_{26}& \Omega_{27}& 0& \Omega_{29}& 0& 0& 0& 0& 0\\ \ast& \ast& \Omega_{33}& 0& \Omega_{35}& \Omega_{36}& \Omega _{37}& 0& 0& 0& 0& 0& 0& 0\\ \ast& \ast& \ast& \Omega_{44}& 0& 0& 0& 0& 0& 0& 0& 0& 0& 0\\ \ast& \ast& \ast& \ast& \Omega_{55}& 0& 0& 0& \Omega_{59}& 0& 0& 0& 0& 0\\ \ast& \ast& \ast& \ast& \ast& \Omega_{66}& 0& 0& \Omega_{69}& 0& 0& 0& 0& 0\\ \ast& \ast& \ast& \ast& \ast& \ast& \Omega_{77}& 0& \Omega _{79}& 0& 0& 0& 0& 0\\ \ast& \ast& \ast& \ast& \ast& \ast& \ast& \Omega_{88}& 0& 0& 0& 0& 0& 0\\ \ast& \ast& \ast& \ast& \ast& \ast& \ast& \ast& \Omega_{99}& 0& 0& 0& 0& 0\\ \ast& \ast& \ast& \ast& \ast& \ast& \ast& \ast& \ast& \Omega _{1010}& 0& 0& 0& 0\\ \ast& \ast& \ast& \ast& \ast& \ast& \ast& \ast& \ast& \ast& \Omega_{1111}& 0& 0& 0\\ \ast& \ast& \ast& \ast& \ast& \ast& \ast& \ast& \ast& \ast& \ast& \Omega_{1212}& 0& 0\\ \ast& \ast& \ast& \ast& \ast& \ast& \ast& \ast& \ast& \ast& \ast& \ast& \Omega_{1313}& 0\\ \ast& \ast& \ast& \ast& \ast& \ast& \ast& \ast& \ast& \ast& \ast& \ast& \ast& \Omega_{1414} \end{array}\displaystyle \right ] \\& \hphantom{\Omega_{j}} < 0, \end{aligned}$$

*where*

$$\begin{aligned}& \Xi_{11} = \lambda_{i}R_{1i}, \qquad \Omega_{11} = \mu _{j}\widetilde{R}_{1j},\qquad \Xi_{22}= -C_{i}H_{i}+\sum _{l=1}^{N}\gamma_{il}H_{l}+ \frac{\lambda _{i}}{1-\tau_{1}}e^{-\eta_{i}\bar{\tau}_{1}}R_{2}+\eta_{i}H_{i}, \\& \Omega_{22} = -D_{j}\widetilde{H}_{j}+\sum _{l=1}^{N}\tilde{\gamma }_{jl} \widetilde{H}_{l}+\frac{\mu_{j}}{1-\tau_{2}}e^{\tilde{-\eta }_{j}\bar{\tau}_{2}} \widetilde{R}_{2}+\widetilde{\eta }_{j}\widetilde{H}_{j}, \qquad \Xi_{33}= \frac{1}{1-\tau_{1}}E^{2}S+ \sigma_{1}K^{2}T, \\& \Omega_{33} = \frac{1}{1-\tau_{2}}\widetilde{E}^{2} \widetilde{S} + \sigma_{2}\widetilde{K}^{2}\widetilde{T}, \qquad \Xi_{44}= -e^{\eta_{i}\bar{\tau}_{1}}S, \qquad \Omega_{26} = \widetilde {H}_{j}V_{1j}, \qquad \Omega_{1212} = - \mu_{j}\widetilde{R}_{2}, \\& \Omega_{44} = -e^{-\widetilde{\eta}_{j}\bar{\tau}_{2}}\widetilde {S}, \qquad \Omega_{55} = N_{2}, \qquad \Xi_{66}= -(1-\tau _{1})e^{-\eta_{i}\bar{\tau}_{1}}N_{3}, \\& \Omega_{66} = -(1-\tau _{2})e^{-\widetilde{\eta}_{j}\bar{\tau}_{2}}N_{4},\qquad \Xi_{88} = -\frac{1}{\sigma_{1}}T,\qquad \Omega_{88} = - \frac {1}{\sigma_{2}}\widetilde{T}, \\& \Xi_{99} = \sum _{l=1}^{N} \gamma_{il}C_{i}^{T}H_{l}C_{i}+ \eta _{i}C_{i}^{T}H_{i}C_{i},\qquad \Xi_{13} = \eta_{i}P-PC_{i},\qquad \Omega_{69} = -D_{j}^{T} \widetilde{H}_{j}V_{1j}, \\& \Omega_{99} = \sum_{l=1}^{N} \tilde{\gamma}_{jl}D_{j}^{T}\widetilde{H}_{l} D_{j}+\widetilde{\eta}_{j}D_{j}^{T} \widetilde{H}_{j}D_{j},\qquad \Omega_{1010} = \mu_{j}\widetilde{R}_{3j}, \qquad \Omega_{37}= QV_{2j}, \\& \Omega_{13} = \tilde{\eta}_{j}Q-QD_{j}, \qquad \Xi_{19} = 0,\qquad \Omega_{19} = 0,\qquad \Xi_{25} = H_{i}W_{0i}, \qquad \Omega_{25} = \widetilde {H}_{j}V_{0j}, \\& \Xi_{26} = H_{i}W_{1i},\qquad \Xi_{27} = H_{i}W_{2i}, \qquad \Omega_{27}= \widetilde {H}_{j}V_{2j}, \\& \Xi_{29} = C_{i}^{T}H_{i}C_{i}- \sum_{l=1}^{N}\gamma _{il}C_{i}H_{l}- \eta_{i}C_{i}^{T}H_{i}, \qquad \Xi_{1212} = -\lambda _{i}R_{2}, \\& \Xi_{35} = PW_{0i}, \qquad\Omega_{35} = QV_{0j}, \qquad \Xi_{36} =PW_{1i}, \qquad \Omega_{36} =QV_{1j}, \qquad \Xi_{37}=PW_{2i}, \\& \Xi_{1010} = \lambda_{i}R_{3i},\qquad \Xi_{59} = -C_{i}^{T}H_{i}W_{0i}, \qquad \Omega_{59} =-D_{j}^{T} \widetilde{H}_{j}V_{0j},\qquad \Xi_{69} = -C_{i}^{T}H_{i}W_{1i}, \\& \Xi_{55}= N_{1},\qquad \Omega_{1111} = \mu_{j}\widetilde{R}_{2j},\qquad \Xi_{79} = -C_{i}^{T}H_{i}W_{2i}, \qquad \Omega_{79} = -D_{j}^{T} \widetilde{H}_{j}V_{2j}, \\& \Omega_{29} = D_{j}^{T}\widetilde {H}_{j}D_{j}-\sum _{l=1}^{N} \tilde{\gamma}_{jl} D_{j} \widetilde {H}_{l}-\widetilde{\eta}_{j}D_{j}^{T} \widetilde{H}_{j},\qquad \Omega_{12} = 0, \qquad \Xi_{1111} = \lambda_{i}R_{2i}, \\& \Xi _{1313} = N_{3}, \qquad \Omega_{1313} = N_{4}, \qquad\Xi_{12}= 0,\qquad \Xi_{1414} = \sigma_{1}N_{5}, \\& \Xi_{77} = -\frac{1}{\sigma_{1}}N_{5},\qquad \Omega_{1414} = \sigma_{2}N_{6},\qquad \Omega_{77} = -\frac{1}{\sigma_{2}}N_{6}. \end{aligned}$$

### Proof

Let us construct the following Lyapunov–Krasovskii functional candidate:

$$ V\bigl(t,u(t),v(t),i,j\bigr) = \sum_{l=1}^{8} V_{l}\bigl(t,u(t),v(t),i,j\bigr), $$

where

$$\begin{aligned}& \begin{aligned} V_{1}\bigl(t,u(t),v(t),i,j\bigr) &= e^{\eta_{i}t} \biggl\{ \biggl[u(t)-C_{i} \int_{t-\nu _{1}}^{t}u(s)\,ds \biggr]^{T}H_{i} \biggl[u(t)-C_{i} \int_{t-\nu _{1}}^{t}u(s)\,ds \biggr] \biggr\} \\ &\quad{}+e^{\widetilde{\eta}_{j}t} \biggl\{ \biggl[v(t)-D_{j} \int_{t-\nu _{2}}^{t}v(s)\,ds \biggr]^{T} \widetilde{H}_{j} \biggl[v(t)-D_{j} \int_{t-\nu _{2}}^{t}v(s)\,ds \biggr] \biggr\} , \end{aligned} \\& \begin{aligned} V_{2}\bigl(t,u(t),v(t),i,j\bigr) &= \frac{\lambda_{i}}{1-\tau_{1}} \int_{t-\tau _{1}(t)}^{t}e^{\eta_{i}(s+\tau_{1}(s))}u^{T}(s)R_{2}u(s) \,ds \\ &\quad{} + \frac{\mu_{j}}{1-\tau_{2}} \int_{t-\tau _{2}(t)}^{t}e^{\widetilde{\eta}_{j}(s+\tau _{2}(s))}v^{T}(s) \widetilde{R}_{2}v(s)\,ds, \end{aligned} \\& \begin{aligned} V_{3}\bigl(t,u(t),v(t),i,j\bigr) &= \frac{1}{1-\tau_{1}} \int_{t-\tau_{1}(t)}^{t}e^{\eta _{i}(s)}\bar{g}^{T} \bigl(u(s)\bigr)S\bar{g}\bigl(u(s)\bigr)\,ds \\ &\quad{} + \frac{1}{1-\tau_{2}} \int_{t-\tau_{2}(t)}^{t}e^{\widetilde {\eta}_{j}s}\bar{ \tilde{g}}^{T}\bigl(v(s)\bigr)\widetilde{S} \bar {\tilde{g}} \bigl(u(s)\bigr)\,ds, \end{aligned} \\& \begin{aligned} V_{4}\bigl(t,u(t),v(t),i,j\bigr) &= \int_{-\sigma_{1}}^{0} \int_{t+s}^{t} e^{\eta_{i}\theta}\bar {h}^{T} \bigl(u(\theta)\bigr)T \bar{h}\bigl(u(\theta)\bigr)\,d\theta\,ds \\ &\quad{} + \int_{-\sigma_{2}}^{0} \int_{t+s}^{t} e^{\widetilde{\eta}_{j}\theta}\bar{ \tilde{h}}^{T}\bigl(v(\theta )\bigr)\widetilde{T} \bar{\tilde{h}} \bigl(v(\theta)\bigr)\,d\theta\,ds, \end{aligned} \\& V_{5}\bigl(t,u(t),v(t),i,j\bigr) = 2e^{\eta_{i}t}\sum _{l=1}^{n}p_{l} \int_{0}^{u_{l}(t)}\bar {f}_{l}(\theta)\,d \theta+ 2e^{\widetilde{\eta}_{j}t}\sum_{l=1}^{n}q_{l} \int_{0}^{v_{l}(t)}\bar{\tilde{f}}_{l}(\theta )\,d\theta, \\& \begin{aligned} V_{6}\bigl(t,u(t),v(t),i,j\bigr) &= \int_{0}^{t} e^{\eta_{i}s} \bar{f}^{T} \bigl(v(s)\bigr)N_{1}\bar {f}\bigl(v(s)\bigr)\,ds \\ &\quad {}+ \int_{0}^{t} e^{\widetilde{\eta}_{j}s}\bar{\tilde {f}}^{T}\bigl(u(s)\bigr)N_{2}\bar{\tilde{f}}\bigl(u(s)\bigr) \,ds, \end{aligned} \\& \begin{aligned} V_{7}\bigl(t,u(t),v(t),i,j\bigr) & = \int_{t-\tau_{1}(t)}^{t} e^{\eta_{i}s} \bar{g}^{T} \bigl(v(s)\bigr) N_{3}\bar{g}\bigl(v(s)\bigr)\,ds \\ &\quad{}+ \int_{t-\tau_{2}(t)}^{t} e^{\widetilde{\eta }_{j}s} \bar{ \tilde{g}}^{T}\bigl(u(s)\bigr) N_{4}\bar{\tilde{g}}\bigl(u(s)\bigr)\,ds, \end{aligned} \\& \begin{aligned} V_{8}\bigl(t,u(t),v(t),i,j\bigr) & = \int_{-\sigma_{1}}^{0} \int_{t+s}^{t} e^{\eta_{i}\theta} \bar{h}^{T} \bigl(v(\theta)\bigr)N_{5} \bar{h}\bigl(v(\theta)\bigr) \,d\theta\,ds \\ &\quad{}+ \int_{-\sigma_{2}}^{0} \int_{t+s}^{t} e^{\widetilde{\eta }_{j}\theta}\bar{\tilde{h}}^{T}\bigl(u(\theta)\bigr) N_{6}\bar{\tilde {h}}\bigl(u(\theta)\bigr) \,d\theta\,ds. \end{aligned} \end{aligned}$$

By Assumption 4, (5) and (6), we obtain

$$\begin{aligned}& \operatorname{trace} \bigl[\bar{\rho}_{1}^{T}(u_{1},v_{1},v_{2},t,i)H_{i} \bar {\rho }_{1}(u_{1},v_{1},v_{2},t,i) \bigr] \leq \lambda_{i}\bigl[ u_{1}^{T}R_{1i}u_{1}+v_{1}^{T}R_{2i}v_{1}+v_{2}^{T}R_{3i}v_{2} \bigr], \\& \operatorname{trace} \bigl[\bar{\rho }_{2}^{T}(v_{1},u_{1},u_{2},t,j) \widetilde{H}_{j} \bar{\rho}_{2}(v_{1},u_{1},u_{2},t,j) \bigr] \leq \mu _{j}\bigl[v_{1}^{T} \widetilde{R}_{1j}v_{1}+u_{1}^{T} \widetilde {R}_{2j}u_{1}+u_{2}^{T} \widetilde{R}_{3j}u_{2}\bigr]. \end{aligned}$$

It is easy to prove that system (4) is equivalent to the following form:

$$\begin{aligned}& d\biggl[u(t)-C_{i} \int_{t-\nu_{1}}^{t}u(s)\,ds\biggr] = \biggl[-C_{i}u(t)+W_{0i} \bar {f}\bigl(v(t) \bigr)+W_{1i}\bar{g}\bigl(v\bigl(t-\tau_{1}(t)\bigr)\bigr) \\& \hphantom{d\biggl[u(t)-C_{i} \int_{t-\nu_{1}}^{t}u(s)\,ds\biggr] ={}}{}+W_{2i} \int_{t-\sigma _{1}}^{t}\bar{h}\bigl(v(s)\bigr)\,ds\biggr]\,dt \\& \hphantom{d\biggl[u(t)-C_{i} \int_{t-\nu_{1}}^{t}u(s)\,ds\biggr] ={}}{}+\bar{\rho}_{1}\bigl(u(t-\nu_{1}),v(t),v\bigl(t- \tau _{1}(t)\bigr),t,i\bigr)\,d\bar{\omega}(t), \\& d\biggl[v(t)-D_{j} \int_{t-\nu_{2}}^{t}v(s)\,ds\biggr] = \biggl[-D_{j}v(t)+V_{0j} \bar {\tilde{f}}\bigl(u(t) \bigr)+V_{1j}\bar{\tilde{g}}\bigl(u\bigl(t-\tau _{2}(t)\bigr) \bigr) \\& \hphantom{ d\biggl[v(t)-D_{j} \int_{t-\nu_{2}}^{t}v(s)\,ds\biggr] ={}}{}+V_{2j} \int_{t-\sigma_{2}}^{t}\bar{\tilde {h}}\bigl(u(s)\bigr)\,ds \biggr]\,dt \\& \hphantom{ d\biggl[v(t)-D_{j} \int_{t-\nu_{2}}^{t}v(s)\,ds\biggr] ={}}{}+\bar{\rho}_{2}\bigl(v(t-\nu_{2}),u(t),u\bigl(t-\tau _{2}(t)\bigr),t,j\bigr)\,d\bar{\tilde{\omega}}(t). \end{aligned}$$

By utilizing Lemmas 1.6 and 1.10, from (4) and Definition 1.4, one has

11 $$\begin{aligned}& \mathcal{L}V_{1} \leq e^{\eta_{i}t} \Biggl\{ u^{T}(t) (-2C_{i}H_{i}+\eta _{i}H_{i})u(t)+2u^{T}(t)H_{i}W_{0i} \bar {f}\bigl(v(t)\bigr)+2u^{T}(t)H_{i}W_{1i} \\& \hphantom{\mathcal{L}V_{1} \leq{}}{}\times\bar{g}\bigl(v\bigl(t-\tau_{1}(t)\bigr) \bigr)+2u^{T}(t) H_{i}W_{2i}\biggl( \int_{t-\sigma_{1}}^{t}\bar{h}\bigl(v(s)\bigr)\,ds\biggr)+2 \biggl( \int_{t-\nu _{1}}^{t}u(s)\,ds\biggr)^{T}C_{i}^{T}H_{i} \\& \hphantom{\mathcal{L}V_{1} \leq{}}{}\times C_{i}u(t-\nu_{1})-2\biggl( \int _{t-\nu _{1}}^{t}u(s)\,ds\biggr)^{T}C_{i}^{T}H_{i} W_{1i}\bar{g}\bigl(v\bigl(t-\tau _{1}(t)\bigr)\bigr)-2 \biggl( \int_{t-\nu_{1}}^{t}u(s)\,ds\biggr)^{T} \\& \hphantom{\mathcal{L}V_{1} \leq{}}{}\times C_{i}^{T}H_{i}W_{2i} \biggl( \int _{t-\sigma _{1}}^{t}\bar{h}\bigl(v(s)\bigr)\,ds \biggr)+u^{T}(t)\sum_{l=1}^{N} \gamma _{il}H_{i}u(t)-2u^{T}(t) \Biggl(\sum _{l=1}^{N}\gamma_{il}C_{i}H_{i} \\& \hphantom{\mathcal{L}V_{1} \leq{}}{}+\eta_{i}C_{i}^{T}H_{i} \Biggr) \biggl( \int_{t-\nu _{1}}^{t}u(s)\,ds\biggr) +\biggl( \int_{t-\nu_{1}}^{t}u(s)\,ds\biggr)^{T}\Biggl( \sum_{l=1}^{N}\gamma_{il}C_{i}^{T}H_{i}C_{i}+ \eta _{i}C_{i}^{T}H_{i}C_{i} \Biggr) \\& \hphantom{\mathcal{L}V_{1} \leq{}}{}\times\biggl( \int_{t-\nu_{1}}^{t}u(s)\, ds\biggr)+u^{T}(t-\nu _{1})\lambda_{i}R_{1i}u(t-\nu_{1})+v^{T}(t) \lambda _{i}R_{2i}v(t)+v^{T}\bigl(t- \tau_{1}(t)\bigr) \\& \hphantom{\mathcal{L}V_{1} \leq{}}{}\times\lambda_{i}R_{3i}v\bigl(t-\tau _{1}(t)\bigr) \Biggr\} + e^{\widetilde{\eta}_{j}t} \Biggl\{ v^{T}(t) (-2D_{j}\widetilde {H}_{j}+\widetilde{\eta}_{j} \widetilde{H}_{j})v(t) +2v^{T}(t)\widetilde{H}_{j}V_{0j} \bar{\tilde{f}}\bigl(u(t)\bigr) \\& \hphantom{\mathcal{L}V_{1} \leq{}}{}+2v^{T}(t)\widetilde {H}_{j}V_{1j} \bar{\tilde {g}}\bigl(u\bigl(t-\tau_{2}(t)\bigr)\bigr)+2v^{T}(t) \widetilde{H}_{j} V_{2j} \biggl( \int _{t-\sigma_{2}}^{t}\bar{\tilde{h}}\bigl(u(s)\bigr)\,ds \biggr) \\& \hphantom{\mathcal{L}V_{1} \leq{}}{}+2\biggl( \int_{t-\nu _{2}}^{t}v(s)\,ds\biggr)^{T} D_{j}^{T} \widetilde {H}_{j}D_{j}v(t-\nu _{2})-2\biggl( \int_{t-\nu_{2}}^{t}v(s)\,ds\biggr)^{T} \\& \hphantom{\mathcal{L}V_{1} \leq{}}{}\times D_{j}^{T} \widetilde {H}_{j}V_{1j} \bar{\tilde{g}}\bigl(u\bigl(t- \tau_{2}(t)\bigr)\bigr) \\& \hphantom{\mathcal{L}V_{1} \leq{}}{}-2\biggl( \int_{t-\nu _{2}}^{t}v(s)\,ds\biggr)^{T}D_{j}^{T} \widetilde{H}_{j}V_{2j}\biggl( \int_{t-\sigma _{2}}^{t}\bar{\tilde{h}}\bigl(u(s)\bigr)\,ds \biggr)+v^{T}(t)\sum_{l=1}^{N} \tilde {\gamma}_{jl}\widetilde{H}_{l}v(t)-2v^{T}(t) \\& \hphantom{\mathcal{L}V_{1} \leq{}}{}\times\Biggl(\sum_{l=1}^{N} \tilde{\gamma }_{jl}D_{j}\widetilde {H}_{j}+ \widetilde{\eta}_{j}D_{j}^{T}\widetilde{H}_{j} \Biggr) \biggl( \int_{t-\nu _{2}}^{t}v(s)\,ds\biggr)+\biggl( \int_{t-\nu_{2}}^{t}v(s)\,ds\biggr)^{T} \Biggl( \sum_{l=1}^{N}\tilde{\gamma}_{jl}D_{j}^{T} \widetilde {H}_{j}D_{j} \\& \hphantom{\mathcal{L}V_{1} \leq{}}{}+\widetilde{\eta }_{j}D_{j}^{T} \widetilde{H}_{j}D_{j}\Biggr) \biggl( \int _{t-\nu_{2}}^{t}v(s)\,ds\biggr)+v^{T}(t- \nu_{2})\mu_{j}\widetilde {R}_{1j}v(t- \nu_{2})+u^{T}(t)\mu_{j}\widetilde{R}_{2j} \\& \hphantom{\mathcal{L}V_{1} \leq{}}{}\times u(t)+u^{T}\bigl(t-\tau_{2}(t)\bigr) \mu _{j}\widetilde {R}_{3j}u\bigl(t-\tau_{2}(t) \bigr) \Biggr\} , \end{aligned}$$

12 $$\begin{aligned}& \mathcal{L}V_{2} \leq\frac{\lambda_{i}}{1-\tau_{1}}e^{\eta_{i}(t-\bar {\tau}_{1})}u^{T}(t)R_{2}u(t) - \lambda_{i}e^{\eta_{i}t}u^{T}\bigl(t-\tau _{1}(t)\bigr)R_{2}u\bigl(t-\tau_{1}(t)\bigr) \\& \hphantom{\mathcal{L}V_{2} \leq{}}{} +\frac{\mu_{j}}{1-\tau _{2}}e^{\widetilde{\eta }_{j}(t-\bar{\tau}_{2})}v^{T}(t) \widetilde{R}_{2}v(t)-\mu _{j}e^{\widetilde{\eta}_{j}t}v^{T} \bigl(t-\tau_{2}(t)\bigr)\widetilde {R}_{2}v(t- \tau_{2}(t), \end{aligned}$$

13 $$\begin{aligned}& \mathcal{L}V_{3} \leq \frac{1}{1-\tau_{1}}e^{\eta _{i}t} \bar{f}^{T}\bigl(u(t)\bigr)E^{2}S\bar{f}(u(t) -e^{\eta_{i}(t-\bar{\tau}_{1})}\bar{g}^{T}\bigl(u\bigl(t-\tau _{1}(t) \bigr)\bigr)S \\& \hphantom{\mathcal{L}V_{3} \leq{}}{} \times\bar{g}\bigl(u\bigl(t-\tau _{1}(t)\bigr) \bigr)+\frac {1}{1-\tau_{2}}e^{\widetilde{\eta}_{j}t}\bar{\tilde {f}}^{T} \bigl(v(t)\bigr)\widetilde{E}^{2}\widetilde{S} \bar{\tilde {f}} \bigl(v(t)\bigr)-e^{\widetilde{\eta}_{j}(t-\bar{\tau}_{2})} \\& \hphantom{\mathcal{L}V_{3} \leq{}}{} \times\bar{\tilde {g}}^{T}\bigl(v\bigl(t-\tau _{2}(t)\bigr)\bigr)\widetilde{S} \bar{\tilde{g}}\bigl(v\bigl(t- \tau _{2}(t)\bigr)\bigr), \end{aligned}$$

14 $$\begin{aligned}& \mathcal{L}V_{4} \leq\sigma_{1}e^{\eta_{i}t} \bar{f}^{T}\bigl(u(t)\bigr)K^{2}T\bar{f}\bigl(u(t)\bigr)- \frac{1}{\sigma_{1}}e^{\eta_{i}t} \biggl( \int_{t-\sigma_{1}}^{t}\bar {h}\bigl(u(s)\bigr)\,ds \biggr)^{T}T \\& \hphantom{\mathcal{L}V_{4} \leq{}}{} \times \biggl( \int_{t-\sigma _{1}}^{t}\bar {h}\bigl(u(s)\bigr)\,ds \biggr)+ \sigma_{2}e^{\tilde{\eta}_{j}t}\bar{\tilde {f}}^{T}\bigl(v(t)\bigr) \widetilde{K}^{2}\widetilde{T}\bar{\tilde {f}}\bigl(v(t)\bigr)- \frac{1}{\sigma_{2}} \\& \hphantom{\mathcal{L}V_{4} \leq{}}{} \times e^{\widetilde{\eta }_{j}t} \biggl( \int_{t-\sigma _{2}}^{t}\bar{\tilde{h}}\bigl(v(s)\bigr)\,ds \biggr)^{T}\widetilde{T} \biggl( \int _{t-\sigma_{2}}^{t}\bar{\tilde{h}}\bigl(v(s)\bigr)\,ds \biggr), \end{aligned}$$

15 $$\begin{aligned}& \mathcal{L}V_{5} \leq2e^{\eta_{i}t}\bar{f}^{T} \bigl(u(t)\bigr) (\eta _{i}P-PC_{i})u(t- \nu_{1})+2e^{\eta_{i}t}\bar{f}^{T}\bigl(u(t) \bigr)PW_{0i}\bar {f}\bigl(v(t)\bigr) \\& \hphantom{\mathcal{L}V_{5} \leq{}}{} +2e^{\eta_{i}t}\bar {f}^{T}\bigl(u(t) \bigr)PW_{1i}\bar {g}\bigl(v\bigl(t-\tau_{1}(t)\bigr) \bigr)+2e^{\eta_{i}t}\bar{f}^{T}\bigl(u(t)\bigr)PW_{2i} \int _{t-\sigma_{1}}^{t}\bar{h}\bigl(v(s)\bigr)\,ds \\& \hphantom{\mathcal{L}V_{5} \leq{}}{} +2e^{\widetilde{\eta}_{j}t}\bar {\tilde {f}}^{T}\bigl(v(t) \bigr) (\widetilde{\eta}_{j}Q-QD_{j})v(t-\nu _{2})+2e^{\widetilde{\eta}_{j}t}\bar{\tilde {f}}^{T}\bigl(v(t) \bigr)QV_{0j}\bar{\tilde{f}}\bigl(u(t)\bigr) \\& \hphantom{\mathcal{L}V_{5} \leq{}}{} +2e^{\widetilde{\eta}_{j}t}\bar {\tilde {f}}^{T}\bigl(v(t) \bigr)QV_{1j}\bar{\tilde{g}}\bigl(u\bigl(t-\tau_{2}(t)\bigr) \bigr) +2e^{\widetilde{\eta}_{j}t}\bar{\tilde{f}}^{T}\bigl(v(t) \bigr)QV_{2j} \int _{t-\sigma_{2}}^{t}\bar{\tilde{h}}\bigl(u(s)\bigr) \,ds, \end{aligned}$$

16 $$\begin{aligned}& \mathcal{L}V_{6} = e^{\eta_{i}t} \bar{f}^{T}\bigl(v(t) \bigr) N_{1} \bar{f}\bigl(v(t)\bigr)+e^{\widetilde{\eta}_{j}t} \bar{ \tilde{f}}^{T}\bigl(u(t)\bigr) N_{2} \bar{\tilde{f}}\bigl(u(t) \bigr), \end{aligned}$$

17 $$\begin{aligned}& \mathcal{L}V_{7} \leq e^{\eta_{i}t} \bar{g}^{T} \bigl(v(t)\bigr) N_{3} \bar{g}\bigl(v(t)\bigr) \\& \hphantom{\mathcal{L}V_{7} \leq{}}{}- e^{\eta_{i}(t-\bar{\tau}_{1})} \bar{g}^{T}\bigl(v\bigl(t-\tau_{1}(t)\bigr) \bigr)N_{3} \bar{g} \bigl(v\bigl(t-\tau_{1}(t)\bigr)\bigr) (1-\tau_{1}) \\& \hphantom{\mathcal{L}V_{7} \leq{}}{} + e^{\widetilde{\eta}_{j}t} \bar {\tilde {g}}^{T}\bigl(u(t) \bigr)N_{4}\bar{\tilde{g}}\bigl(u(t)\bigr) \\& \hphantom{\mathcal{L}V_{7} \leq{}}{}- e^{\widetilde{\eta }_{j}(t-\bar{\tau}_{2})} \bar{ \tilde{g}}^{T}\bigl(u\bigl(t-\tau _{2}(t)\bigr) \bigr)N_{4}\bar{\tilde{g}}\bigl(u\bigl(t-\tau_{2}(t)\bigr) \bigr) (1-\tau_{2}), \end{aligned}$$

18 $$\begin{aligned}& \mathcal{L}V_{8} \leq\sigma_{1} e^{\eta_{i}t} \bar{h}^{T}\bigl(v(t)\bigr) N_{5} \bar {h}\bigl(v(t)\bigr)- \frac{1}{\sigma_{1}}e^{\eta_{i}t} \biggl( \int_{t-\sigma _{1}}^{t}\bar{h}\bigl(v(s)\bigr)\,ds \biggr)^{T}N_{5} \biggl( \int_{t-\sigma _{1}}^{t}\bar{h}\bigl(v(s)\bigr)\,ds \biggr) \\& \hphantom{\mathcal{L}V_{8} \leq{}}{} + \sigma_{2} e^{\widetilde{\eta }_{j}t}\bar{\tilde{h}}^{T}\bigl(u(t)\bigr) N_{6} \bar{\tilde {h}}\bigl(u(t)\bigr) \\& \hphantom{\mathcal{L}V_{8} \leq{}}{}-\frac{1}{\sigma_{2}}e^{\widetilde{\eta}_{j}t} \biggl( \int _{t-\sigma_{2}}^{t}\bar{\tilde{h}}\bigl(u(s)\bigr)\,ds \biggr)^{T}N_{6} \biggl( \int _{t-\sigma_{2}}^{t}\bar{\tilde{h}}\bigl(u(s)\bigr)\,ds \biggr). \end{aligned}$$

By combining Eqs. (11)–(18), we can obtain that

19 $$ \mathcal{L}V\bigl(t,u(t),v(t),i,j\bigr) \leq e^{\eta_{i}t}\Psi ^{T}(t)\Xi_{i}\Psi(t)+e^{\widetilde{\eta}_{j}t}\Phi^{T}(t) \Omega_{j}\Phi(t), $$

where

$$\begin{aligned} \begin{aligned} \Psi(t) & = \biggl[u^{T}(t-\nu_{1}),u^{T}(t),f^{T} \bigl(u(t)\bigr),\bar {g}^{T}\bigl(u\bigl(t-\tau_{1}(t)\bigr) \bigr), \bar{f} ^{T}\bigl(v(t)\bigr),\bar{g}^{T}\bigl(v \bigl(t-\tau _{1}(t)\bigr)\bigr), \\ &\quad \biggl( \int_{t-\sigma_{1}}^{t}\bar{h}\bigl(v(s)\bigr)\,ds \biggr)^{T}, \biggl( \int_{t-\sigma_{1}}^{t}\bar{h}\bigl(u(s)\bigr)\,ds \biggr)^{T}, \biggl( \int _{t-\nu_{1}}^{t}u(s)\,ds \biggr)^{T}, v^{T}\bigl(t-\tau_{1}(t)\bigr), \\ &\quad v^{T}(t), u^{T}\bigl(t-\tau_{1}(t)\bigr), \bar{g}\bigl(v(t)\bigr),\bar {h}\bigl(v(t)\bigr) \biggr] \end{aligned} \end{aligned}$$

and

$$\begin{aligned} \Phi(t) =& \biggl[v^{T}(t-\nu_{2}),v^{T}(t),\bar{ \tilde {f}} ^{T}\bigl(v(t)\bigr), \bar{\tilde{g}}^{T}\bigl(v \bigl(t-\tau_{2}(t)\bigr)\bigr),\bar{\tilde{f}}^{T}\bigl(u(t) \bigr), \bar{\tilde{g}}^{T}\bigl(u\bigl(t-\tau_{2}(t)\bigr) \bigr), \\ & \biggl( \int_{t-\sigma_{2}}^{t}\bar{\tilde{h}}\bigl(u(s)\bigr)\,ds \biggr)^{T}, \biggl( \int_{t-\sigma_{2}}^{t} \bar{\tilde{h}}\bigl(v(s)\bigr)\,ds \biggr)^{T}, \biggl( \int_{t-\nu _{2}}^{t}v(s)\,ds \biggr)^{T}, u^{T}\bigl(t-\tau_{2}(t)\bigr), \\ & u^{T}(t), v^{T}\bigl(t-\tau_{2}(t)\bigr),\bar{ \tilde {g}}^{T}\bigl(u(t)\bigr),\bar{\tilde{h}}^{T}\bigl(u(t) \bigr) \biggr]. \end{aligned}$$

Let $\alpha= \min_{i\in\mathcal {S}}\lambda_{\min}(-\Xi_{i})$ and $\beta= \min_{j\in\mathcal {S}}\mu_{\min}(-\Omega_{j})$. From conditions (9) and (10), it is easy to see that $\alpha>0$ and $\beta>0$. This fact together with (19) gives

20 $$ \mathcal{L}V\bigl(t,u(t),v(t),i,j\bigr) \leq -\bigl(\alpha e^{\eta_{i}t} \bigl( \bigl\Vert u(t) \bigr\Vert ^{2}+ \bigl\Vert v(t) \bigr\Vert ^{2}\bigr)+\beta e^{\widetilde{\eta}_{j}t}\bigl( \bigl\Vert u(t) \bigr\Vert ^{2}+ \bigl\Vert v(t) \bigr\Vert ^{2}\bigr)\bigr). $$

Then, for $t = t_{k}$, by some simple calculations, one gets

$$ V_{1}\bigl(t_{k},u(t_{k}),v(t_{k}),i,j \bigr) - V_{1}\bigl(t_{k^{-}},u(t_{k^{-}}),v(t_{k^{-}}),i,j \bigr) < 0. $$

Therefore $V_{1}(t_{k},u(t_{k}),v(t_{k}),i,j) \leq V_{1}(t_{k^{-}},u(t_{k^{-}}),v(t_{k^{-}}),i,j)$, $k\in\mathbb{Z}_{+}$, which implies that $V(t_{k},u(t_{k}),v(t_{k}),i,j) \leq V(t_{k^{-}},u(t_{k^{-}}),v(t_{k^{-}}),i,j)$, $k\in\mathbb{Z}_{+}$. Using mathematical induction, we have that, for all $i,j \in\mathfrak {M}$ and $k \geq1 $,

$$\begin{aligned} \mathbb{E}V\bigl(t_{k},u(t_{k}),v(t_{k}),i,j \bigr) \leq& \mathbb {E}V\bigl(t_{k^{-}},u(t_{k^{-}}),v(t_{k^{-}}),i,j \bigr) \\ \leq& \mathbb{E}V\bigl(t_{k-1},u(t_{k-1}),v(t_{k-1}),r(t_{k-1}), \tilde {r}(t_{k-1})\bigr) \\ \leq& \mathbb{E}V\bigl(t_{k-1}^{-},u\bigl(t_{k-1}^{-} \bigr),v\bigl(t_{k-1}^{-}\bigr),r\bigl(t_{k-1}^{-} \bigr), \tilde{r}\bigl(t_{k-1}^{-}\bigr)\bigr) \\ \leq& \mathbb{E}V\bigl(t_{0},u(t_{0}),v(t_{0}),r(t_{0}), \tilde {r}(t_{0})\bigr). \end{aligned}$$

Since $t>t'$, it follows from *Dynkin’s* formula that we have

$$\begin{aligned}& \mathbb{E}V\bigl(t,u(t),v(t),i,j\bigr) \leq \mathbb {E}V\bigl(t',u \bigl(t'\bigr),v\bigl(t'\bigr),r\bigl(t' \bigr),\tilde{r}\bigl(t'\bigr)\bigr), \\& \mathbb{E}V\bigl(t,u(t),v(t),i,j\bigr) \leq \mathbb {E}V\bigl(0,u(0),v(0),r(0), \tilde{r}(0)\bigr). \end{aligned}$$

Hence it follows from the definition of $V(t,u(t),v(t),i,j)$, the generalized *Ito’s* formula, and (20) that

21 $$\begin{aligned} \begin{aligned}[b] &\lambda_{\min}(H_{i}) \mathbb{E} {\bigl( \bigl\Vert u(t) \bigr\Vert ^{2}+ \bigl\Vert v(t) \bigr\Vert ^{2}\bigr)}+ \mu _{\min}(\widetilde{H}_{j}) \mathbb{E} {\bigl( \bigl\Vert u(t) \bigr\Vert ^{2}+ \bigl\Vert v(t) \bigr\Vert ^{2} \bigr)} \\ &\quad \leq e^{-\eta_{i}t}{\mathbb{E}V\bigl(0,u(0),v(0),r(0),\tilde {r}(0) \bigr)}+e^{-\eta_{i}t} {\mathbb{E}\biggl[ \int_{0}^{t}-\alpha e^{\eta _{i}t}\bigl( \bigl\Vert u(s) \bigr\Vert ^{2}+ \bigl\Vert v(s) \bigr\Vert ^{2}\bigr)\,ds\biggr]} \\ &\qquad{}+e^{-\tilde{\eta}_{j}t}{\mathbb {E}V\bigl(0,u(0),v(0),r(0),\tilde{r}(0)\bigr)} +e^{-\widetilde{\eta}_{j}t}{\mathbb{E}\biggl[ \int_{0}^{t}-\beta e\widetilde{\eta}_{j}t \bigl( \bigl\Vert u(s) \bigr\Vert ^{2}+ \bigl\Vert v(s) \bigr\Vert ^{2}\bigr)\,ds\biggr]} \\ &\quad \leq e^{-\eta_{i}t}{\mathbb{E}V\bigl(0,u(0),v(0),r(0),\tilde{r}(0) \bigr)} +e^{-\widetilde{\eta}_{j}t}{\mathbb{E}V\bigl(0,u(0),v(0),r(0),\tilde {r}(0)\bigr).} \end{aligned} \end{aligned}$$

By (21), we can get that $\lim_{t\rightarrow+\infty}\mathbb {E}{(\|u(t)\|^{2}+\|v(t)\|^{2})} = 0 $ and $\lim_{t\rightarrow+\infty }\mathbb{E}(\|u(t)\|^{2}+ \|v(t)\|^{2}) = 0$. Furthermore,

22 $$ \begin{aligned} &\lim_{t\rightarrow+\infty}\mathbb {E} {\bigl( \bigl\vert u_{l}(t) \bigr\vert ^{2}+ \bigl\vert v_{l}(t) \bigr\vert ^{2}\bigr)} = 0 \quad \mbox{and} \\ &\lim_{t\rightarrow+\infty}\mathbb{E} {\bigl( \bigl\vert u_{m}(t) \bigr\vert ^{2}+ \bigl\vert v_{m}(t) \bigr\vert ^{2}\bigr)} = 0, \quad l,m = 1,2, \ldots,n. \end{aligned} $$

For $\bar{f}_{l}(\theta)$ and $\bar{\tilde{f}}_{m}(\theta)$, by Lemma 1.6 there exist constants $q_{l0}>0$, $\tilde{q}_{m0}>0$, and $r_{l0}>0$, $\tilde{r}_{m0}>0$ such that $|\bar{f}_{l}(\theta)| \geq q_{l0}|\theta|^{\alpha}$, $\forall |\theta| \leq r_{l0}$, $l=1,2,\ldots,n$, and $|\bar{\tilde{f}}_{m}(\theta) |\geq\tilde{q}_{m0}|\theta |^{\alpha}$, $\forall |\theta| \leq\tilde{r}_{m0}$, $m=1,2,\ldots,n$.

By (22), there exists a scalar $\mathcal{T}>0$, when $t\geq\mathcal{T}$, $\mathbb{E}\{u_{l}(t)\} \in[-\bar {r}_{0},\bar{r}_{0}]$, $l=1,2,\ldots,n$, where $\bar{r}_{0} = \min_{1\leq l \leq n} r_{l0}$ and $\mathbb{E}\{ v_{m}(t)\} \in[-\bar{\tilde{r}}_{0},\bar{\tilde{r}}_{0}]$, $m = 1,2,\ldots,n$, where $\bar{\tilde{r}}_{0} = \min_{1 \leq l \leq n}\tilde{r}_{m0}$. Hence when $t\geq\mathcal{T}$, one gets

23 $$\begin{aligned}& e^{-\eta_{i}t} {\mathbb{E}V\bigl(0,u(0),v(0),r(0)\bigr)}+e^{-\widetilde{\eta }_{j}t}{ \mathbb{E}V\bigl(0,u(0),v(0),\tilde{r}(0)\bigr)} \\& \quad \geq\frac{2pq_{0}}{\alpha+1} \Bigl\{ \max_{1\leq l \leq n} \mathbb {E} \bigl\{ \bigl\Vert u_{l}(t) \bigr\Vert ^{2} \bigr\} \Bigr\} ^{\frac{\alpha+1}{2}} +\frac{2\tilde{p}\tilde{q}_{0}}{\alpha+1} \Bigl\{ \max_{1\leq m \leq n} \mathbb{E} \bigl\{ \bigl\Vert v_{m}(t) \bigr\Vert ^{2} \bigr\} \Bigr\} ^{\frac{\alpha +1}{2}}, \end{aligned}$$

where $p = \min_{1\leq l \leq n}p_{l}$, $\tilde{p} = \min_{1\leq m \leq n}\tilde{p}_{m}$ and $q_{0} = \min_{1 \leq l \leq n}q_{l0}$, $\tilde{q}_{0} = \min_{1 \leq m \leq n}\tilde{q}_{m0}$. By (23), we get

$$\begin{aligned}& \Bigl\{ \max_{1\leq l \leq n} \mathbb{E} \bigl\{ \bigl\Vert u_{l}(t) \bigr\Vert ^{2} \bigr\} \Bigr\} ^{\frac{\alpha+1}{2}}+ \Bigl\{ \max_{1\leq m \leq n} \mathbb{E} \bigl\{ \bigl\Vert v_{m}(t) \bigr\Vert ^{2} \bigr\} \Bigr\} ^{\frac{\alpha+1}{2}} \\& \quad \leq \biggl(\frac{\alpha+1}{2pq_{0}} \biggr)^{*} \bigl\{ \bigl( \mathbb {E}V\bigl(0,u(0),v(0),r(0),\tilde{r}(0)\bigr)\bigr)e^{-\eta t}\bigr\} \\& \qquad \biggl(\mbox{Put } \biggl(\frac{\alpha+1}{2pq_{0}}\biggr)^{*} = \max\biggl\{ \frac {\alpha+1}{2pq_{0}},\frac{\alpha+1}{2\tilde{p}\tilde{q}_{0}}\biggr\} \mbox{ and } \eta= \min_{i,j\in\mathfrak{M}}\Bigl\{ \min_{i\in\mathfrak {M}} \eta_{i},\min_{j\in\mathfrak{M}}\tilde{\eta}_{j}\Bigr\} \biggr) , \end{aligned}$$

where

24 $$\begin{aligned} \mathbb{E}V\bigl(0,u(0),v(0),r(0),\tilde{r}(0)\bigr) \leq& \lambda_{\max}(H_{i})\mathbb{E} \Vert \xi \Vert ^{2}+\mu_{\max }(\widetilde{H}_{j})\mathbb{E} \Vert \tilde{\xi} \Vert ^{2}+\lambda_{\max }(R_{2}) \frac{\lambda_{i}}{1-\tau_{1}} \\ &{} \times\frac{e^{\eta_{i}\bar{\tau}_{1}}-1}{\eta_{i}(1+\tau _{1})}\mathbb{E} \Vert \xi \Vert ^{2}+ \mu_{\mathrm{max}}(\widetilde{R}_{2}) \frac{\mu_{j}}{1-\tau_{2}} \frac{e^{\widetilde{\eta}_{j}\bar {\tau}_{2}}-1}{\widetilde{\eta}_{j}(1+\tau_{2})} \\ &{} \times\mathbb{E} \Vert \tilde{\xi} \Vert ^{2}+ \lambda_{\max}(S)\frac {1}{1-\tau_{1}} \frac{1-e^{\eta_{i}\bar{\tau}_{1}}}{ \eta_{i}}\mathbb{E} \bigl\Vert \bar{g}(\xi) \bigr\Vert ^{2}+\mu_{\max }(\widetilde {S}) \\ &{} \times\frac{1}{1-\tau_{2}} \frac{1-e^{-\widetilde{\eta }_{j}\bar{\tau}_{2}}}{\widetilde{\eta}_{j}}\mathbb{E} \bigl\Vert \bar { \tilde{g}}(\tilde{\xi}) \bigr\Vert ^{2} +\lambda_{\max}(T) \sigma_{1}\frac {1-e^{-\eta_{i}t}}{\eta_{i}} \\ &{} \times\mathbb{E} \bigl\Vert \bar{h}(\xi) \bigr\Vert ^{2}+ \mu_{\max}(\widetilde{T})\sigma_{2}\frac {1-e^{-\widetilde{\eta}_{j}t}}{\widetilde{\eta}_{j}} \mathbb{E} \bigl\Vert \bar{\tilde{h}}(\tilde{\xi}) \bigr\Vert ^{2} \\ &{} +2P \mathbb{E} \bigl\vert \xi\bar{f}(\xi ) \bigr\vert +2Q\mathbb{E} \bigl\vert \tilde{\xi}\bar{\tilde{f}}(\tilde{\xi}) \bigr\vert . \end{aligned}$$

Let

25 $$ \pi_{\xi_{i}+\tilde{\xi}_{j}} = (\pi_{\xi_{i}}+\pi_{\tilde{\xi_{j}}})^{\frac{2}{\alpha +1}}, $$

where

$$\begin{aligned}& \pi_{\xi_{i}} = n \biggl\{ \biggl(\frac{\alpha+1}{2pq_{0}} \biggr)^{*}\biggl(\lambda_{\max}(H_{i})\mathbb{E} \Vert \xi \Vert ^{2}+\lambda _{\max }(R_{2}) \frac{\lambda_{i}}{1-\tau_{1}} \frac{e^{\eta_{i}\bar{\tau}_{1}}-1}{\eta_{i}(1+\tau_{1})}\mathbb {E} \Vert \xi \Vert ^{2} \\& \hphantom{\pi_{\xi_{i}} ={}}{}+\lambda_{\max}(S)\frac{1}{1-\tau_{1}}\frac{1-e^{\eta_{i}\bar{\tau }_{1}}}{\eta_{i}}\mathbb {E} \bigl\Vert \bar{g}(\xi) \bigr\Vert ^{2} \\& \hphantom{\pi_{\xi_{i}} ={}}{}+\lambda_{\max}(T)\sigma_{1} \frac {1-e^{-\eta_{i}\bar{\tau}_{1}}}{ \eta_{i}}\mathbb{E} \bigl\Vert \bar{h}(\xi) \bigr\Vert ^{2}+2P\mathbb{E} \bigl\vert \xi\bar {f}(\xi) \bigr\vert \biggr) \biggr\} , \\& \pi_{\tilde{\xi}_{j}} = n \biggl\{ \biggl(\frac{\alpha +1}{2pq_{0}} \biggr)^{*}\biggl( \mu_{\max}(\widetilde{H}_{j}) \mathbb{E} \Vert \tilde{\xi} \Vert ^{2}+\mu_{\max}( \widetilde{R}_{2}) \frac{\mu_{j}}{1-\tau_{2}} \frac{e^{-\widetilde{\eta}_{j}\bar {\tau}_{2}}-1}{ \widetilde{\eta}_{j}(1+\tau_{2})}\mathbb{E} \Vert \tilde{\xi} \Vert ^{2} \\& \hphantom{\pi_{\tilde{\xi}_{j}} ={}}{}+\mu_{\max}(\widetilde{S}) \frac{1}{1-\tau_{2}}\frac{1-e^{-\widetilde{\eta }_{j}\bar{\tau }_{2}}}{\widetilde{\eta}_{j}}\mathbb{E} \bigl\Vert \bar{ \tilde{g}}(\tilde {\xi}) \bigr\Vert ^{2} \\& \hphantom{\pi_{\tilde{\xi}_{j}} ={}}{}+\mu_{\max}( \widetilde{T})\sigma_{2}\frac {1-e^{-\widetilde{\eta}_{j}\bar{\tau}_{2}}}{\widetilde{\eta }_{j}}\mathbb{E} \bigl\Vert \bar{ \tilde{h}}(\tilde{\xi}) \bigr\Vert ^{2} +2Q\mathbb {E} \bigl\vert \tilde{\xi}\bar{\tilde{f}}(\tilde{\xi}) \bigr\vert \biggr) \biggr\} . \end{aligned}$$

Let $\chi= \max_{i,j\in\mathfrak{M}}\{\pi_{\xi_{i}+\tilde {\xi}_{j}}\}$. It follows from (23), (24), and (25) that

$$ \mathbb{E}\bigl\{ \bigl\Vert u(t) \bigr\Vert ^{2}\bigr\} + \mathbb{E}\bigl\{ \bigl\Vert v(t) \bigr\Vert ^{2}\bigr\} \leq \chi e^{-\frac{2\eta}{\alpha+1}t}. $$

Therefore

26 $$ \mathbb{E}\bigl\{ \bigl\Vert u(t) \bigr\Vert ^{2}\bigr\} + \mathbb{E}\bigl\{ \bigl\Vert v(t) \bigr\Vert ^{2}\bigr\} \leq \chi e^{-\frac{2\eta}{\alpha+1}t} \quad \mbox{for all } t>0. $$

By Definition 1.3 and (26), we see that the equilibrium point of neural networks (4) is globally exponentially stable in the mean square sense. □

### Remark 2.2

To the best of our knowledge, the global exponential stability criteria for impulsive effects of SBAMNNs with Markovian jump parameters and mixed time, leakage term delays, *α*-inverse Holder activation functions have not been discussed in the existing literature. Hence this paper reports a new idea and some sufficient conditions for global exponential stability conditions of neural networks, which generalize and improve the outcomes in [9, 11, 21, 37, 38].

### Remark 2.3

The criteria given in Theorem 2.1 are dependent on the time delay. It is well known that the delay-dependent criteria are less conservative than the delay-independent criteria, particularly when the delay is small. Based on Theorem 2.1, the following result can be obtained easily.

### Remark 2.4

If there are no stochastic disturbances in system (4), then the neural networks are simplified to

27 $$\begin{aligned} \begin{aligned} &du(t) = \biggl[-C\bigl(r(t)\bigr)u(t- \nu_{1})+W_{0}\bigl(r(t)\bigr)\bar {f}\bigl(v(t) \bigr) \\ &\hphantom{du(t) ={}}{}+W_{1}\bigl(r(t)\bigr) \bigl(\bar{g}\bigl(v\bigl(t- \tau_{1}(t)\bigr)\bigr)\bigr) \\ &\hphantom{du(t) ={}}{}+W_{2}\bigl(r(t)\bigr) \int_{t-\sigma_{1}}^{t}\bar{h}\bigl(v(s)\bigr)\,ds\biggr]\, dt, \quad t>0, t \neq t_{k}, \\ &\Delta u(t_{k}) = \bar{M}_{k}\bigl(u\bigl(t_{k}^{-} \bigr),u_{t_{k}^{-}}\bigr),\quad t=t_{k}, k\in\mathbb{Z}_{+}, \\ &dv(t) = \biggl[-D\bigl(\tilde{r}(t)\bigr)v(t-\nu_{2})+V_{0} \bigl(\tilde{r}(t)\bigr)\bar {\tilde{f}}\bigl(u(t)\bigr) \\ &\hphantom{dv(t) ={}}{}+V_{1}\bigl( \tilde{r}(t)\bigr)\bar{\tilde{g}}\bigl(u\bigl(t-\tau _{2}(t)\bigr) \bigr) \\ &\hphantom{dv(t) ={}}{}+V_{2}\bigl(\tilde{r}(t)\bigr) \int_{t-\sigma_{2}}^{t}\bar{\tilde {h}}\bigl(u(s)\bigr)\,ds \biggr]\,dt,\quad t>0, t \neq t_{k}, \\ &\Delta v(t_{k}) = \bar{N}_{k}\bigl(v\bigl(t_{k}^{-} \bigr),v_{t_{k}^{-}}\bigr), \quad t=t_{k}, k\in\mathbb{Z}_{+}. \end{aligned} \end{aligned}$$

## Global exponential stability of uncertain system

Now consider the following BAM neural networks with stochastic noise disturbance, Markovian jump parameters, leakage and mixed time delays, which are in the uncertainty case system:

28 $$\begin{aligned} \begin{aligned} &du(t) = \biggl[-\bigl(C+\Delta C(t)\bigr) \bigl(r(t) \bigr)u(t-\nu_{1}) \\ &\hphantom{du(t) ={}}{}+\bigl(W_{0}+\Delta W_{0}(t) \bigr) \bigl(r(t)\bigr)\bar{f}\bigl(v(t)\bigr) \\ &\hphantom{du(t) ={}}{}+\bigl(W_{1}+W_{1}(t) \bigr) \bigl(r(t)\bigr)\bigl(\bar{g}\bigl(v\bigl(t-\tau_{1}(t)\bigr) \bigr)\bigr) \\ &\hphantom{du(t) ={}}{}+\bigl(W_{2}+\Delta W_{2}(t)\bigr) \bigl(r(t) \bigr) \int_{t-\sigma_{1}}^{t}\bar{h}\bigl(v(s)\bigr)\,ds\biggr]\,dt \\ &\hphantom{du(t) ={}}{}+ \bar{\rho _{1}}\bigl(u(t-\nu_{1}),v(t),v\bigl(t-\tau_{1}(t)\bigr),t,r(t)\bigr)\,d\bar{ \omega}(t), \quad t>0, t \neq t_{k}, \\ &\Delta u(t_{k}) = \bigl(\bar{M}_{k}+\Delta\bar {M}_{k}(t)\bigr) \bigl(r(t)\bigr) \bigl(u\bigl(t_{k}^{-} \bigr),u_{t_{k}^{-}}\bigr),\quad t=t_{k}, k\in \mathbb {Z}_{+}, \\ &dv(t) = \biggl[-\bigl(D+\Delta D(t)\bigr) \bigl(\tilde{r}(t)\bigr)v(t- \nu_{2}) \\ &\hphantom{dv(t) = {}}{}+\bigl(V_{0}+\Delta V_{0}(t)\bigr) \bigl( \tilde{r}(t)\bigr)\bar{\tilde{f}}\bigl(u(t)\bigr) \\ &\hphantom{dv(t) = {}}{}+\bigl(V_{1}+\Delta V_{1}(t)\bigr) \bigl(\tilde{r}(t)\bigr)\bar{\tilde{g}}\bigl(u\bigl(t-\tau _{2}(t) \bigr)\bigr) \\ &\hphantom{dv(t) = {}}{}+\bigl(V_{2}+\Delta V_{2}(t)\bigr) \bigl( \tilde{r}(t)\bigr) \int_{t-\sigma_{2}}^{t}\bar{\tilde {h}}\bigl(u(s)\bigr)\,ds \biggr]\,dt \\ &\hphantom{dv(t) = {}}{}+\bar{\rho_{2}}\bigl(v(t-\nu_{2}),u(t),u\bigl(t-\tau_{2}(t)\bigr),t,\tilde{r}(t)\bigr) \,d \bar{\tilde {\omega }}(t), \quad t>0, t \neq t_{k}, \\ &\Delta v(t_{k}) = \bigl(\bar{N}_{k}+\Delta \bar{N}_{k}(t)\bigr) \bigl(\tilde {r}(t)\bigr) \bigl(v(t_{k^{-}}),v_{t_{k^{-}}} \bigr), \quad t = t_{k}, k\in\mathbb {Z}_{+}. \end{aligned} \end{aligned}$$

### Assumption 5

The perturbed uncertain matrices $\Delta C(t)$, $\Delta D(t)$, $\Delta W_{0i}(t)$, $\Delta W_{1i}(t)$, $\Delta W_{2i}(t)$, $\Delta V_{0j}(t)$, $\Delta V_{1j}(t)$, and $\Delta V_{2j}(t)$ are time-varying functions satisfying: $\Delta W_{0i}(t) = MF_{l}(t)N_{W_{0i}}$, $\Delta W_{1i}(t) = MF_{l}(t)N_{W_{1i}}$, $\Delta W_{2i}(t) = MF_{l}(t)N_{W_{2i}}$, $\Delta V_{0j}(t) = MF_{l}(t)N_{V_{0j}}$, $\Delta V_{1j}(t) = MF_{l}(t)N_{V_{1j}}$, $\Delta V_{2j}(t) = MF_{l}(t)N_{V_{2j}}$, $\Delta C(t) = MF_{l}(t)N_{C_{i}}$ and $\Delta D(t)=MF_{l}(t)N_{D_{j}}$, where *M*, $N_{W_{0i}}$, $N_{W_{1i}}$, $N_{W_{2i}}$, $N_{V_{0j}}$, $N_{V_{1j}}$, $N_{V_{2j}}$, $N_{C_{i}}$, and $N_{D_{j}}$ are given constant matrices, respectively. $F_{l_{z}}(t)$ ($l = 0, 1, 2, 3$) (where $z= \mbox{either } i \mbox{ or } j$) are unknown real time-varying matrices which have the following structure: $F_{l_{z}}(t)= \operatorname{blockdiag}\{\delta _{l_{1}}(t)I_{z_{l_{1}}},\ldots,\delta _{l_{k}}(t)I_{z_{l_{k}}}, F_{l_{1}}(t), \ldots,F_{l_{s}}(t)\}$, $\delta_{l_{z}} \in\mathbb {R}$, $|\delta_{l_{z}}|\leq1$, $1 \leq z \leq\tilde{k}$ and $F_{l_{p}}^{T} F_{l_{p}}\leq I$, $1\leq p \leq s$. We define the set $\Delta_{l}$ as $\Delta_{l} = \{ F_{l_{z}}^{T}(t)F_{l_{z}}(t)\leq I, F_{l_{z}}N_{l_{z}} = N_{l_{z}}F_{l_{z}}, \forall N_{l_{z}} \in\Gamma_{l_{z}}\} $, where $\Gamma_{l_{z}} = \{N_{l_{z}}= \operatorname{blockdiag}[N_{l_{1}},\ldots ,N_{l_{k}},n_{l_{1}}I_{f_{l_{1}}}, \ldots, n_{l_{s}}I_{f_{l_{s}}}]\}$, $N_{l_{z}}$ invertible for $1 \leq z \leq\tilde{k}$ and $n_{l_{p}} \in\mathbb{R}$, $n_{l_{p}} \neq0$, for $1 \leq p \leq s$ and *p*, *k̃*, $s \in \mathfrak{M}$.

Also $\Delta H_{i}$, $\Delta\widetilde{H}_{j}$, $\Delta R_{1i}$, $\Delta R_{2i}$, $\Delta R_{3i}$, $\Delta\widetilde{R}_{1j}$, $\Delta \widetilde{R}_{2j}$, $\Delta\widetilde{R}_{3j}$, $\Delta R_{2}$, $\Delta\widetilde{R}_{2}$, Δ*S*, Δ*T*, Δ*S̃*, Δ*T̃*, $\Delta N_{1}$, $\Delta N_{2}$, $\Delta N_{3}$, $\Delta N_{4}$, $\Delta N_{5}$, and $\Delta N_{6}$ are positive definite diagonal matrices that are defined as follows: $\Delta H_{i} = \check{E} \Sigma F_{H_{i}}$, $\Delta\widetilde{H}_{j} = \check{E} \Sigma F_{\widetilde{H}_{j}}$ and $\Delta R_{1i} = \check{E} \Sigma F_{R_{1i}}$, where *Ě*, $F_{H_{i}}$, $F_{\widetilde{H}_{j}}$, $F_{R_{1i}}$, $F_{R_{2i}}$, $F_{R_{3i}}$, $F_{\widetilde{R}_{1j}}$, $F_{\widetilde{R}_{2j}}$, $F_{\widetilde{R}_{3j}}$, $F_{R_{2}}$, $F_{\widetilde{R}_{2}}$, $F_{S}$, $F_{\widetilde{S}}$, $F_{T}$, $F_{\widetilde{T}}$, $F_{N_{1}}$, $F_{N_{2}}$, $F_{N_{3}}$, $F_{N_{4}}$, $F_{N_{5}}$, and $F_{N_{6}}$ are positive diagonal matrices (i.e., $F_{H_{i}}F_{H_{i}}^{T} = \operatorname{diag}(h_{1}^{*},h_{2}^{*},\ldots,h_{n}^{*})$, $F_{\widetilde {H}_{j}}F_{\widetilde{H}_{j}}^{T} = \operatorname{diag}(\tilde {h}_{1}^{*}, \tilde {h}_{2}^{*},\ldots,\tilde{h}_{n}^{*})$, where $h_{i}^{*}, \tilde {h}_{j}^{*} > 0$ ($i,j = 1,2,\ldots,n$)) and the remaining terms are defined in a similar way, which characterizes how the deterministic uncertain parameter in Σ enters the nominal matrices $H_{i}$, $\widetilde{H}_{j}$, $R_{b_{i}}$ ($b = 1, 2, 3$), $\widetilde {R}_{c_{j}}$ ($c = 1, 2, 3$), *S*, *S̃*, *T*, *T̃*, $N_{1}$, $N_{2}$, $N_{3}$, $N_{4}$, $N_{5}$, and $N_{6}$. The matrix Σ with real entries, which may be time-varying, is unknown and satisfies $\Sigma^{T} \Sigma\leq I$.

### Remark 3.1

Overall, the stability of time-delayed neural networks fully depends on the Lyapunov–Krasovskii functional and LMI concepts. In particular, based on the neural networks, different types of LKF are chosen or handled to lead to the system stability. Up to now, no one has considered uncertain parameters in Lyapunov–Krasovskii functional terms. Without loss of generality, the gap is initially filled in this work, and also this kind of approach gives more advanced and less conserved stability results.

### Theorem 3.2

*Under Assumptions* 1, 2, *and* 5, *the neural network system* (28) *is global robust exponentially stable in the mean square if*, *for given* $\eta_{i}, \widetilde{\eta}_{j}>0$ ($i,j\in \mathfrak{M}$), *there exist positive definite matrices* *S*, *T*, *S̃*, *T̃*, $R_{2}$, $\widetilde{R}_{2}$, $N_{1}$, $N_{2}$, $N_{3}$, $N_{4}$, $N_{5}$, $N_{6}$ *and* $H_{i}$, $\widetilde{H}_{j}$ ($i,j\in \mathfrak{M}$), *positive definite diagonal matrices* Δ*S*, Δ*T*, $\Delta{R}_{2}$, Δ*S̃*, Δ*T̃*, $\Delta \widetilde{R}_{2}$, $\Delta H_{i}$, $\Delta\widetilde{H}_{j}$, $\Delta N_{1}$, $\Delta N_{2}$, $\Delta N_{3}$, $\Delta N_{4}$, $\Delta N_{5}$, $\Delta N_{6}$, *P*, *Q* *and positive scalars* $\lambda_{i}$ *and* $\mu_{j}$ ($i,j\in\mathfrak{M}$) *such that the following LMIs are satisfied*:

29 $$\begin{aligned}& (H_{i}+\Delta H_{i}) < \lambda_{i}I, \end{aligned}$$

30 $$\begin{aligned}& (\widetilde{H}_{j}+ \Delta\widetilde{H}_{j}) < \mu_{j}J, \end{aligned}$$

31 $$\begin{aligned}& (\bar{M}_{k}+\Delta\bar{M}_{k})^{T}(H_{i}+ \Delta H_{i}) (\bar {M}_{k}+\Delta\bar{M}_{k}) - (H_{j}+\Delta H_{j}) \leq 0, \end{aligned}$$

32 $$\begin{aligned}& (\bar{N}_{k}+\Delta\bar{N}_{k})^{T}( \widetilde{H}_{i}+\Delta \widetilde{H}_{i}) ( \bar{N}_{k}+\Delta\bar{N}_{k}) - (\widetilde {H}_{j}+\Delta\widetilde{H}_{j}) \\& \quad \leq 0\quad \bigl[ \textit{here } r(t_{k})=i \textit{ and } \tilde{r}(t_{k})=j \bigr], \end{aligned}$$

33 $$\begin{aligned}& \Omega^{*} = \left [ \textstyle\begin{array}{@{}c@{\quad}c@{\quad}c@{\quad}c@{\quad}c@{\quad}c@{}} \Xi_{1}^{*}& \Xi_{2}^{*}& \Xi_{3}^{*}& \Xi_{4}^{*}& \Xi_{5}^{*}& \Xi_{10}^{*}\\ \ast& \Xi_{6}^{*}& 0& 0& 0& 0\\ \ast& \ast& \Xi_{7}^{*}& 0& 0& 0\\ \ast& \ast& \ast& \Xi_{8}^{*}& 0& 0\\ \ast& \ast& \ast& \ast& \Xi_{9}^{*}& 0\\ \ast& \ast& \ast& \ast& \ast& \Xi_{11}^{*} \end{array}\displaystyle \right ] < 0, \end{aligned}$$

34 $$\begin{aligned}& \Pi= \left [ \textstyle\begin{array}{@{}c@{\quad}c@{\quad}c@{\quad}c@{\quad}c@{\quad}c@{}} \Delta_{1}^{*}& \Delta_{2}^{*}& \Delta_{3}^{*}& \Delta_{4}^{*}& \Delta_{5}^{*}& \Delta_{10}^{*}\\ \ast& \Delta_{6}^{*}& 0& 0& 0& 0\\ \ast& \ast& \Delta_{7}^{*}& 0& 0& 0\\ \ast& \ast& \ast& \Delta_{8}^{*}& 0& 0\\ \ast& \ast& \ast& \ast& \Delta_{9}^{*}& 0\\ \ast& \ast& \ast& \ast& \ast& \Delta_{11}^{*} \end{array}\displaystyle \right ] < 0, \end{aligned}$$

*where*

$$\begin{aligned}& \Xi_{6}^{*} = \operatorname{diag}\{\Theta_{i} \}, \qquad \Xi_{7}^{*} = \operatorname{diag}\{\Theta _{l_{1}}\}, \qquad \Delta_{6}^{*} = \operatorname{diag}\{\Phi_{j}\}, \qquad \Delta _{7}^{*} = \operatorname{diag}\{\Phi_{l_{2}}\}, \\ & \quad i, j = 1,2,3,\ldots,12 \textit{ and } l_{1}, l_{2} = 13,14,15,\ldots,24; \\ & \Xi_{8}^{*} = \operatorname{diag}\{\tilde{ \Theta}_{s}\}, \qquad \Xi _{9}^{*} = \operatorname{diag}\{\tilde{\Theta}_{l_{3}}\}, \qquad \Delta_{8}^{*} = \operatorname{diag}\{\tilde { \Phi}_{s^{*}}\}, \qquad \Delta_{9}^{*} = \operatorname{diag}\{\tilde {\Phi }_{l_{4}}\}, \\ & \quad s, s^{*} = 1,2,3,\ldots,12 \textit{ and } l_{3}, l_{4} = 13,14,15,16; \\ & \Xi_{11}^{*} = \operatorname{diag}\bigl\{ \Theta_{l_{5}}^{*}\bigr\} ,\qquad \Delta _{11}^{*} = \operatorname{diag}\bigl\{ \Phi_{l_{6}}^{*}\bigr\} , \quad l_{5}, l_{6} = 1,2,3,\ldots,10; \end{aligned}$$

$$ \Xi_{1}^{*} = \left [ \textstyle\begin{array}{@{}c@{\quad}c@{\quad}c@{\quad}c@{\quad}c@{\quad}c@{\quad }c@{\quad}c@{\quad}c@{\quad}c@{\quad}c@{\quad}c@{\quad}c@{\quad}c@{}} \Xi_{11}& \Xi_{12}& \Xi_{13}& 0& 0& 0& 0& 0& \Xi_{19}& 0& 0& 0& 0& 0\\ \ast& \Xi_{22}& 0& 0& \Xi_{25}& \Xi_{26}& \Xi_{27}& 0& \Xi _{29}^{*}& 0& 0& 0& 0& 0\\ \ast& \ast& \Xi_{33}& 0& \Xi_{35}& \Xi_{36}& \Xi_{37}& 0& 0& 0& 0& 0& 0& 0\\ \ast& \ast& \ast& \Xi_{44}& 0& 0& 0& 0& 0& 0& 0& 0& 0& 0\\ \ast& \ast& \ast& \ast& \Xi_{55}& 0& 0& 0& \Xi_{59}& 0& 0& 0& 0& 0\\ \ast& \ast& \ast& \ast& \ast& \Xi_{66}& 0& 0& \Xi_{69}^{*}& 0& 0& 0& 0& 0\\ \ast& \ast& \ast& \ast& \ast& \ast& \Xi_{77}& 0& \Xi_{79}^{*}& 0& 0& 0& 0& 0\\ \ast& \ast& \ast& \ast& \ast& \ast& \ast& \Xi_{88}& 0& 0& 0& 0& 0& 0\\ \ast& \ast& \ast& \ast& \ast& \ast& \ast& \ast& \Xi_{99}^{*}& 0& 0& 0& 0& 0\\ \ast& \ast& \ast& \ast& \ast& \ast& \ast& \ast& \ast& \Xi _{1010}& 0& 0& 0& 0\\ \ast& \ast& \ast& \ast& \ast& \ast& \ast& \ast& \ast& \ast& \Xi_{1111}& 0& 0& 0\\ \ast& \ast& \ast& \ast& \ast& \ast& \ast& \ast& \ast& \ast& \ast& \Xi_{1212}& 0& 0\\ \ast& \ast& \ast& \ast& \ast& \ast& \ast& \ast& \ast& \ast& \ast& \ast& \Xi_{1313}& 0\\ \ast& \ast& \ast& \ast& \ast& \ast& \ast& \ast& \ast& \ast& \ast& \ast& \ast& \Xi_{1414} \end{array}\displaystyle \right ], $$

$$ \Delta_{1}^{*} = \left [ \textstyle\begin{array}{@{}c@{\quad}c@{\quad}c@{\quad}c@{\quad}c@{\quad}c@{\quad }c@{\quad}c@{\quad}c@{\quad}c@{\quad}c@{\quad}c@{\quad}c@{\quad}c@{}} \Omega_{11}& \Omega_{12}& \Omega_{13}& 0& 0& 0& 0& 0& \Omega_{19}& 0& 0& 0& 0& 0\\ \ast& \Omega_{22}& 0& 0& \Omega_{25}& \Omega_{26}& \Omega_{27}& 0& \Omega_{29}^{*}& 0& 0& 0& 0& 0\\ \ast& \ast& \Omega_{33}& 0& \Omega_{35}& \Omega_{36}& \Omega _{37}& 0& 0& 0& 0& 0& 0& 0\\ \ast& \ast& \ast& \Omega_{44}& 0& 0& 0& 0& 0& 0& 0& 0& 0& 0\\ \ast& \ast& \ast& \ast& \Omega_{55}& 0& 0& 0& \Omega_{59}& 0& 0& 0& 0& 0\\ \ast& \ast& \ast& \ast& \ast& \Omega_{66}& 0& 0& \Omega _{69}^{*}& 0& 0& 0& 0& 0\\ \ast& \ast& \ast& \ast& \ast& \ast& \Omega_{77}& 0& \Omega _{79}^{*}& 0& 0& 0& 0& 0\\ \ast& \ast& \ast& \ast& \ast& \ast& \ast& \Omega_{88}& 0& 0& 0& 0& 0& 0\\ \ast& \ast& \ast& \ast& \ast& \ast& \ast& \ast& \Omega _{99}^{*}& 0& 0& 0& 0& 0\\ \ast& \ast& \ast& \ast& \ast& \ast& \ast& \ast& \ast& \Omega _{1010}& 0& 0& 0& 0\\ \ast& \ast& \ast& \ast& \ast& \ast& \ast& \ast& \ast& \ast& \Omega_{1111}& 0& 0& 0\\ \ast& \ast& \ast& \ast& \ast& \ast& \ast& \ast& \ast& \ast& \ast& \Omega_{1212}& 0& 0\\ \ast& \ast& \ast& \ast& \ast& \ast& \ast& \ast& \ast& \ast& \ast& \ast& \Omega_{1313}& 0\\ \ast& \ast& \ast& \ast& \ast& \ast& \ast& \ast& \ast& \ast& \ast& \ast& \ast& \Omega_{1414} \end{array}\displaystyle \right ], $$

$$ \Xi_{2}^{*} = \left [ \textstyle\begin{array}{@{}c@{\quad}c@{\quad}c@{\quad}c@{\quad}c@{\quad}c@{\quad }c@{\quad}c@{\quad}c@{\quad}c@{\quad}c@{\quad}c@{}} 0& 0& 0& 0& 0& 0& 0& 0& 0& 0& 0& 0\\ N_{C_{i}}^{T}& \Gamma_{1}& \vartheta_{2}^{T}& \epsilon_{2}\check {E}& \Gamma_{2}& \epsilon_{3}M\check{E}& 0& 0& 0& 0& \hat{\vartheta }_{1}^{T}& 0\\ 0& 0& 0& 0& 0& 0& 0& 0& 0& 0& 0& 0\\ 0& 0& 0& 0& 0& 0& 0& 0& 0& 0& 0& 0\\ N_{W_{0i}}^{T}& 0& \Gamma_{3}& 0& \Gamma_{4}& 0& 0& 0& 0& 0& 0& 0\\ N_{W_{1i}}^{T}& 0& \Gamma_{5}& 0& \Gamma_{6}& 0& \Gamma_{7}& 0& \hat {\vartheta}_{5}^{T}& 0& \hat{\vartheta}_{2}^{T}& 0\\ N_{W_{2i}}^{T}& 0& \Gamma_{8}& 0& \Gamma_{9}& 0& \Gamma_{10}& 0& \hat{\vartheta}_{6}^{T}& 0& \hat{\vartheta}_{3}^{T}& 0\\ 0& 0& 0& 0& 0& 0& 0& 0& 0& 0& 0& 0\\ \vartheta_{1}^{T}& 0& \vartheta_{3}^{T}& 0& \vartheta_{4}^{T}& 0& \vartheta_{5}^{T}& \epsilon_{4}C^{T}\check{E}& \vartheta_{6}^{T}& \epsilon_{5}M^{T}H_{i}& \vartheta_{7}^{T}& \epsilon_{6}M^{T}\check {E}\\ 0& 0& 0& 0& 0& 0& 0& 0& 0& 0& 0& 0\\ 0& 0& 0& 0& 0& 0& 0& 0& 0& 0& 0& 0\\ 0& 0& 0& 0& 0& 0& 0& 0& 0& 0& 0& 0\\ 0& 0& 0& 0& 0& 0& 0& 0& 0& 0& 0& 0\\ 0& 0& 0& 0& 0& 0& 0& 0& 0& 0& 0& 0 \end{array}\displaystyle \right ], $$

$$ \Xi_{3}^{*} = \left [ \textstyle\begin{array}{@{}c@{\quad}c@{\quad}c@{\quad}c@{\quad}c@{\quad}c@{\quad }c@{\quad}c@{\quad}c@{\quad}c@{\quad}c@{\quad}c@{}} 0& 0& 0& 0& 0& 0& -N_{C_{i}}^{T}& 0& 0& 0& 0& 0\\ 0& 0& 0& 0& 0& 0& 0& 0& 0& 0& 0& 0\\ 0& 0& 0& 0& \hat{\vartheta}_{4}^{T}& \epsilon_{9}\check{E}& 0& \epsilon_{10}PM& 0& 0& 0& 0\\ 0& 0& 0& 0& 0& 0& 0& 0& \frac{1}{2}F_{S}^{T}& \epsilon_{11}\check {E}& 0& 0\\ 0& 0& 0& 0& 0& 0& N_{W_{0i}}^{T}& 0& 0& 0& 0& 0\\ \Gamma_{11}& 0& 0& 0& 0& 0& N_{W_{1i}}^{T}& 0& 0& 0& 0& 0\\ \Gamma_{12}& 0& 0& 0& 0& 0& N_{W_{2i}}^{T}& 0& 0& 0& 0& 0\\ 0& 0& 0& 0& 0& 0& 0& 0& 0& 0& \Gamma_{13}& \epsilon_{12}\check{E}\\ \vartheta_{8}^{T}& \epsilon_{7}M\check{E}& 0& 0& 0& 0& 0& 0& 0& 0& 0& 0\\ 0& 0& 0& 0& 0& 0& 0& 0& 0& 0& 0& 0\\ 0& 0& 0& 0& 0& 0& 0& 0& 0& 0& 0& 0\\ 0& 0& \Gamma_{14}& \epsilon_{8}\check{E}& 0& 0& 0& 0& 0& 0& 0& 0\\ 0& 0& 0& 0& 0& 0& 0& 0& 0& 0& 0& 0\\ 0& 0& 0& 0& 0& 0& 0& 0& 0& 0& 0& 0 \end{array}\displaystyle \right ], $$

$$ \Delta_{2}^{*} = \left [ \textstyle\begin{array}{@{}c@{\quad}c@{\quad}c@{\quad}c@{\quad}c@{\quad}c@{\quad }c@{\quad}c@{\quad}c@{\quad}c@{\quad}c@{\quad}c@{}} 0& 0& 0& 0& 0& 0& 0& 0& 0& 0& 0& 0\\ N_{D_{j}}^{T}& \tilde{\Gamma}_{1}& \tilde{\vartheta}_{2}^{T}& \tilde{\epsilon}_{2}\check{E}& \tilde{\Gamma}_{2}& \tilde {\epsilon}_{3}M\check{E}& 0& 0& 0& 0& \hat{\tilde{\vartheta }}_{1}^{T}& 0\\ 0& 0& 0& 0& 0& 0& 0& 0& 0& 0& 0& 0\\ 0& 0& 0& 0& 0& 0& 0& 0& 0& 0& 0& 0\\ N_{V_{0j}}^{T}& 0& \tilde{\Gamma}_{3}& 0& \tilde{\Gamma}_{4}& 0& 0& 0& 0& 0& 0& 0\\ N_{V_{1j}}^{T}& 0& \tilde{\Gamma}_{5}& 0& \tilde{\Gamma}_{6}& 0& \tilde{\Gamma}_{7}& 0& \hat{\tilde{\vartheta}}_{5}^{T}& 0& \hat {\tilde{\vartheta}}_{2}^{T}& 0\\ N_{V_{2j}}^{T}& 0& \tilde{\Gamma}_{8}& 0& \tilde{\Gamma}_{9}& 0& \tilde{\Gamma}_{10}& 0& \hat{\tilde{\vartheta}}_{6}^{T}& 0& \hat {\tilde{\vartheta}}_{3}^{T}& 0\\ 0& 0& 0& 0& 0& 0& 0& 0& 0& 0& 0& 0\\ \tilde{\vartheta}_{1}^{T}& 0& \tilde{\vartheta}_{3}^{T}& 0& \tilde {\vartheta}_{4}^{T}& 0& \tilde{\vartheta}_{5}^{T}& \tilde{\epsilon }_{4}D^{T}\check{E}& \tilde{\vartheta}_{6}^{T}& \tilde{\epsilon }_{5}M^{T}\widetilde{H}_{j}& \tilde{\vartheta}_{7}^{T}& \tilde {\epsilon}_{6}M^{T}\check{E}\\ 0& 0& 0& 0& 0& 0& 0& 0& 0& 0& 0& 0\\ 0& 0& 0& 0& 0& 0& 0& 0& 0& 0& 0& 0\\ 0& 0& 0& 0& 0& 0& 0& 0& 0& 0& 0& 0\\ 0& 0& 0& 0& 0& 0& 0& 0& 0& 0& 0& 0\\ 0& 0& 0& 0& 0& 0& 0& 0& 0& 0& 0& 0 \end{array}\displaystyle \right ], $$

$$ \Delta_{3}^{*} = \left [ \textstyle\begin{array}{@{}c@{\quad}c@{\quad}c@{\quad}c@{\quad}c@{\quad}c@{\quad }c@{\quad}c@{\quad}c@{\quad}c@{\quad}c@{\quad}c@{}} 0& 0& 0& 0& 0& 0& -N_{D_{j}}^{T}& 0& 0& 0& 0& 0\\ 0& 0& 0& 0& 0& 0& 0& 0& 0& 0& 0& 0\\ 0& 0& 0& 0& \hat{\tilde{\vartheta}}_{4}^{T}& \tilde{\epsilon }_{9}\check{E}& 0& \tilde{\epsilon}_{10}Q M& 0& 0& 0& 0\\ 0& 0& 0& 0& 0& 0& 0& 0& \frac{1}{2}F_{\widetilde{S}}^{T}& \tilde {\epsilon}_{11}\check{E}& 0& 0\\ 0& 0& 0& 0& 0& 0& N_{V_{0j}}^{T}& 0& 0& 0& 0& 0\\ \tilde{\Gamma}_{11}& 0& 0& 0& 0& 0& N_{V_{1j}}^{T}& 0& 0& 0& 0& 0\\ \tilde{\Gamma}_{12}& 0& 0& 0& 0& 0& N_{V_{2j}}^{T}& 0& 0& 0& 0& 0\\ 0& 0& 0& 0& 0& 0& 0& 0& 0& 0& \tilde{\Gamma}_{13}& \tilde{\epsilon }_{12}\check{E}\\ \tilde{\vartheta}_{8}^{T}& \tilde{\epsilon}_{7}M\check{E}& 0& 0& 0& 0& 0& 0& 0& 0& 0& 0\\ 0& 0& 0& 0& 0& 0& 0& 0& 0& 0& 0& 0\\ 0& 0& 0& 0& 0& 0& 0& 0& 0& 0& 0& 0\\ 0& 0& \tilde{\Gamma}_{14}& \tilde{\epsilon}_{8}\check{E}& 0& 0& 0& 0& 0& 0& 0& 0\\ 0& 0& 0& 0& 0& 0& 0& 0& 0& 0& 0& 0\\ 0& 0& 0& 0& 0& 0& 0& 0& 0& 0& 0& 0 \end{array}\displaystyle \right ], $$

$$ \Xi_{4}^{*} = \left [ \textstyle\begin{array}{@{}c@{\quad}c@{\quad}c@{\quad}c@{\quad}c@{\quad}c@{\quad }c@{\quad}c@{\quad}c@{\quad}c@{\quad}c@{\quad}c@{}} 0& 0& 0& 0& 0& 0& 0& 0& 0& 0& 0& 0\\ FH_{i}^{T}& \epsilon_{13}\check{E}& 0& \epsilon_{14}M& 0& \Gamma _{4}^{*}& 0& 0& 0& 0& 0& 0\\ 0& 0& 0& 0& 0& 0& 0& 0& 0& 0& 0& 0\\ 0& 0& 0& 0& 0& 0& 0& 0& 0& 0& 0& 0\\ 0& 0& 0& 0& 0& 0& 0& 0& 0& 0& 0& 0\\ 0& 0& 0& 0& 0& 0& 0& 0& 0& 0& 0& 0\\ 0& 0& 0& 0& 0& 0& 0& 0& 0& 0& 0& 0\\ 0& 0& 0& 0& 0& 0& 0& 0& 0& 0& 0& 0\\ \Gamma_{1}^{*}& 0& \Gamma_{2}^{*}& 0& \Gamma_{3}^{*}& 0& \vartheta _{1}^{*{T}}& \epsilon_{16}\check{E}& \Gamma_{5}^{*}& \Gamma _{6}^{*}& \vartheta_{2}^{*{T}}& \Gamma_{7}^{*}\\ 0& 0& 0& 0& 0& 0& 0& 0& 0& 0& 0& 0\\ 0& 0& 0& 0& 0& 0& 0& 0& 0& 0& 0& 0\\ 0& 0& 0& 0& 0& 0& 0& 0& 0& 0& 0& 0\\ 0& 0& 0& 0& 0& 0& 0& 0& 0& 0& 0& 0\\ 0& 0& 0& 0& 0& 0& 0& 0& 0& 0& 0& 0 \end{array}\displaystyle \right ], $$

$$ \Delta_{4}^{*} = \left [ \textstyle\begin{array}{@{}c@{\quad}c@{\quad}c@{\quad}c@{\quad}c@{\quad}c@{\quad }c@{\quad}c@{\quad}c@{\quad}c@{\quad}c@{\quad}c@{}} 0& 0& 0& 0& 0& 0& 0& 0& 0& 0& 0& 0\\ F\widetilde{H}_{j}^{T}& \tilde{\epsilon}_{13}\check{E}& 0& \tilde {\epsilon}_{14}M& 0& \tilde{\Gamma}_{4}^{*}& 0& 0& 0& 0& 0& 0\\ 0& 0& 0& 0& 0& 0& 0& 0& 0& 0& 0& 0\\ 0& 0& 0& 0& 0& 0& 0& 0& 0& 0& 0& 0\\ 0& 0& 0& 0& 0& 0& 0& 0& 0& 0& 0& 0\\ 0& 0& 0& 0& 0& 0& 0& 0& 0& 0& 0& 0\\ 0& 0& 0& 0& 0& 0& 0& 0& 0& 0& 0& 0\\ 0& 0& 0& 0& 0& 0& 0& 0& 0& 0& 0& 0\\ \tilde{\Gamma}_{1}^{*}& 0& \tilde{\Gamma}_{2}^{*}& 0& \tilde {\Gamma}_{3}^{*}& 0& \tilde{\vartheta}_{1}^{*{T}}& \tilde{\epsilon }_{16}\check{E}& \tilde{\Gamma}_{5}^{*}& \tilde{\Gamma}_{6}^{*}& \tilde{\vartheta}_{2}^{*{T}}& \tilde{\Gamma}_{7}^{*}\\ 0& 0& 0& 0& 0& 0& 0& 0& 0& 0& 0& 0\\ 0& 0& 0& 0& 0& 0& 0& 0& 0& 0& 0& 0\\ 0& 0& 0& 0& 0& 0& 0& 0& 0& 0& 0& 0\\ 0& 0& 0& 0& 0& 0& 0& 0& 0& 0& 0& 0\\ 0& 0& 0& 0& 0& 0& 0& 0& 0& 0& 0& 0 \end{array}\displaystyle \right ], $$

$$ \Xi_{5}^{*} = \left [ \textstyle\begin{array}{@{}c@{\quad}c@{\quad}c@{\quad}c@{}} 0& 0& 0& 0\\ 0& 0& 0& 0\\ 0& 0& 0& 0\\ 0& 0& 0& 0\\ 0& 0& 0& 0\\ 0& 0& 0& 0\\ 0& 0& 0& 0\\ 0& 0& 0& 0\\ H_{i}^{T}N_{C_{i}}^{T}& \epsilon_{19}C_{i}^{T}M& \Gamma_{8}^{*}& C_{i}^{T}\check{E}\\ 0& 0& 0& 0\\ 0& 0& 0& 0\\ 0& 0& 0& 0\\ 0& 0& 0& 0\\ 0& 0& 0& 0 \end{array}\displaystyle \right ],\qquad \Delta_{5}^{*} = \left [ \textstyle\begin{array}{@{}c@{\quad}c@{\quad}c@{\quad}c@{}} 0& 0& 0& 0\\ 0& 0& 0& 0\\ 0& 0& 0& 0\\ 0& 0& 0& 0\\ 0& 0& 0& 0\\ 0& 0& 0& 0\\ 0& 0& 0& 0\\ 0& 0& 0& 0\\ \widetilde{H}_{j}^{T}N_{D_{j}}^{T}& \tilde{\epsilon}_{19}D_{j}^{T}M& \tilde{\Gamma}_{8}^{*}& D_{j}^{T}\check{E}\\ 0& 0& 0& 0\\ 0& 0& 0& 0\\ 0& 0& 0& 0\\ 0& 0& 0& 0\\ 0& 0& 0& 0 \end{array}\displaystyle \right ], $$

$$ \Xi_{10}^{*} = \left [ \textstyle\begin{array}{@{}c@{\quad}c@{\quad}c@{\quad}c@{\quad}c@{\quad}c@{\quad }c@{\quad}c@{\quad}c@{\quad}c@{}} 0& 0& 0& 0& 0& 0& 0& 0& 0& 0\\ 0& 0& 0& 0& 0& 0& 0& 0& 0& 0\\ 0& 0& 0& 0& 0& 0& 0& 0& 0& 0\\ 0& 0& 0& 0& 0& 0& 0& 0& 0& 0\\ \epsilon_{21}\check{E}& F_{N_{1}}^{T}& 0& 0& 0& 0& 0& 0& 0& 0\\ 0& 0& \epsilon_{22}\check{E}& \alpha^{*}F_{N_{3}}^{T}& 0& 0& 0& 0& 0& 0\\ 0& 0& 0& 0& \epsilon_{23}\check{E}& \Gamma_{15}& 0& 0& 0& 0\\ 0& 0& 0& 0& 0& 0& 0& 0& 0& 0\\ 0& 0& 0& 0& 0& 0& 0& 0& 0& 0\\ 0& 0& 0& 0& 0& 0& 0& 0& 0& 0\\ 0& 0& 0& 0& 0& 0& 0& 0& 0& 0\\ 0& 0& 0& 0& 0& 0& 0& 0& 0& 0\\ 0& 0& 0& 0& 0& 0& \epsilon_{24}\check{E}& F_{N_{3}}^{T}& 0& 0\\ 0& 0& 0& 0& 0& 0& 0& 0& \epsilon_{25}\check{E}& \sigma_{1}F_{N_{5}}^{T} \end{array}\displaystyle \right ], $$

$$ \Delta_{10}^{*} = \left [ \textstyle\begin{array}{@{}c@{\quad}c@{\quad}c@{\quad}c@{\quad}c@{\quad}c@{\quad }c@{\quad}c@{\quad}c@{\quad}c@{}} 0& 0& 0& 0& 0& 0& 0& 0& 0& 0\\ 0& 0& 0& 0& 0& 0& 0& 0& 0& 0\\ 0& 0& 0& 0& 0& 0& 0& 0& 0& 0\\ 0& 0& 0& 0& 0& 0& 0& 0& 0& 0\\ \tilde{\epsilon}_{21}\check{E}& F_{N_{2}}^{T}& 0& 0& 0& 0& 0& 0& 0& 0\\ 0& 0& \tilde{\epsilon}_{22}\check{E}& \beta^{*}F_{N_{4}}^{T}& 0& 0& 0& 0& 0& 0\\ 0& 0& 0& 0& \tilde{\epsilon}_{23}\check{E}& \tilde{\Gamma}_{15}& 0& 0& 0& 0\\ 0& 0& 0& 0& 0& 0& 0& 0& 0& 0\\ 0& 0& 0& 0& 0& 0& 0& 0& 0& 0\\ 0& 0& 0& 0& 0& 0& 0& 0& 0& 0\\ 0& 0& 0& 0& 0& 0& 0& 0& 0& 0\\ 0& 0& 0& 0& 0& 0& 0& 0& 0& 0\\ 0& 0& 0& 0& 0& 0& \tilde{\epsilon}_{24}\check{E}& F_{N_{4}}^{T}& 0& 0\\ 0& 0& 0& 0& 0& 0& 0& 0& \tilde{\epsilon}_{25}\check{E}& \sigma _{2}F_{N_{6}}^{T} \end{array}\displaystyle \right ], $$

*where*

$$\begin{aligned}& \Theta_{1} =\Theta_{2} =-\epsilon_{1}I,\qquad \Theta_{3}=\Theta _{4}=-\epsilon_{2}I, \qquad \Theta_{7}=\Theta_{8}=-\epsilon _{4}I, \qquad \Theta_{11}=\Theta_{12}=-\epsilon_{6}I, \\& \Theta _{19}=-\epsilon_{10}I,\qquad \Theta_{9} =\Theta_{10}=-\epsilon_{5}I, \qquad \Theta_{13}=\Theta _{14}=-\epsilon_{7}I, \qquad \Theta_{15}=\Theta_{16}=-\epsilon _{8}I, \\& \Theta_{17}=\Theta_{18}=-\epsilon_{9}I, \qquad \Theta _{20}=-\epsilon_{10}I,\qquad \Theta_{5} =\Theta_{6}=-\epsilon_{3}I, \qquad \Theta_{21}=\Theta _{22}=-\epsilon_{11}I, \\& \Theta_{23}=\Theta_{24}=-\epsilon _{12}I,\qquad \Phi_{1}=\Phi_{2}=\tilde{\epsilon}_{1}I, \qquad \Phi_{3}=\Phi _{4}=\tilde{\epsilon}_{2}I,\qquad \Phi_{5} =\Phi_{6}=\tilde{\epsilon}_{3}I, \\& \Phi_{7}=\Phi _{8}=\tilde{\epsilon}_{4}I, \qquad \Phi_{15}=\Phi_{16}=\tilde {\epsilon}_{8}I, \qquad\Phi_{11}=\Phi_{12}=\tilde{\epsilon}_{6}I, \qquad \Phi_{13}=\Phi_{14}=\tilde{\epsilon}_{7}I, \\& \Phi_{9} =\Phi_{10}=\tilde{\epsilon}_{5}I, \qquad \Phi_{17}=\Phi _{18}=\tilde{\epsilon}_{9}I, \qquad \Phi_{19}=\Phi_{20}=\tilde {\epsilon}_{10}I, \qquad \Phi_{21}=\Phi_{22}=\tilde{\epsilon }_{11}I, \\& \Phi_{23}=\Phi_{24}=\tilde{\epsilon}_{12}I,\qquad \Theta_{1}^{*} =\Theta_{2}^{*}=- \epsilon_{21}I, \qquad \Theta _{3}^{*}= \Theta_{4}^{*}=-\epsilon_{22}I, \qquad \Theta_{5}^{*}=\Theta _{6}^{*}=- \epsilon_{23}I, \\& \Theta_{7}^{*}= \Theta_{8}^{*}=-\epsilon_{24}I,\qquad \Theta _{9}^{*}=\Theta_{10}^{*}=- \epsilon_{25}I,\qquad \Phi_{1}^{*} =\Phi_{2}^{*}=-\tilde{ \epsilon}_{21}I, \qquad \Phi _{3}^{*}= \Phi_{4}^{*}=-\tilde{\epsilon}_{22}I, \\& \Phi _{5}^{*}=\Phi_{6}^{*}=-\tilde{ \epsilon}_{23}I,\qquad \Phi _{7}^{*}= \Phi_{8}^{*}=-\tilde{\epsilon}_{24}I,\qquad \Phi _{9}^{*}=\Phi_{10}^{*}=-\tilde{ \epsilon}_{25}I,\qquad \Gamma_{1} = \epsilon_{1}M H_{i}, \\& \Gamma_{2}= N_{C_{i}}^{T}F_{H_{i}}^{T}, \qquad \Gamma_{3}= F_{H_{i}}^{T}W_{0i}^{T}, \Gamma_{4}= F_{H_{i}}^{T}N_{W_{0i}}^{T}, \qquad \Gamma_{5} = F_{H_{i}}^{T} W_{1i}^{T}, \qquad\Gamma_{6} = F_{H_{i}}^{T}N_{W_{1i}}^{T}, \\& \Gamma_{7} = -W_{1}^{T}F_{H_{i}}^{T}, \qquad \Gamma_{8} = F_{H_{i}}^{T} W_{2i}^{T},\qquad \Gamma_{12}= CF_{H_{i}}^{T}N_{W_{2i}}^{T},\qquad \Gamma_{10}= -W_{2}^{T}F_{H_{i}}^{T}, \\& \Gamma_{11}= CF_{H_{i}}^{T}N_{W_{1i}}^{T},\qquad \Gamma_{9} = F_{H_{i}}^{T}N_{W_{2i}}^{T}, \qquad \Gamma_{13}= \frac {-1}{2\sigma_{1}}F_{T}^{T}, \qquad \Gamma_{14} = \frac{-\lambda _{i}}{2}F_{R_{2}}^{T}, \\& \tilde{\Gamma}_{1}= \tilde{\epsilon}_{1}M \widetilde{H}_{j},\qquad \tilde{\Gamma}_{2}= N_{D_{j}}^{T}F_{\widetilde{H}_{j}}^{T}, \qquad \tilde{ \Gamma}_{3}= F_{\widetilde{H}_{j}}^{T}V_{0j}^{T},\qquad \tilde{\Gamma}_{4} = F_{\widetilde{H}_{j}}^{T}N_{V_{0j}}^{T}, \qquad \tilde{\Gamma}_{5} = F_{\widetilde{H}_{j}}^{T} V_{1j}^{T}, \\& \tilde{\Gamma}_{6} = F_{\widetilde{H}_{j}}^{T}N_{V_{1j}}^{T},\qquad \tilde{ \Gamma}_{7} = -V_{1}^{T}F_{\widetilde{H}_{j}}^{T}, \qquad \tilde{\Gamma}_{8} = F_{\widetilde{H}_{j}}^{T} V_{2j}^{T},\qquad \tilde{\Gamma}_{10}= -V_{2}^{T}F_{\widetilde{H}_{j}}^{T}, \\& \tilde{\Gamma}_{9} = F_{\widetilde{H}_{j}}^{T}N_{V_{2j}}^{T}, \qquad \tilde{\Gamma}_{11}= DF_{\widetilde{H}_{j}}^{T}N_{V_{1j}}^{T}, \qquad \tilde{\Gamma}_{12}= DF_{\widetilde{H}_{j}}^{T}N_{V_{2j}}^{T}, \qquad \tilde{\Gamma}_{13}= \frac{-1}{2\sigma_{2}}F_{\widetilde{T}}^{T}, \\& \tilde{\Gamma}_{14} = \frac{-\mu_{j}}{2}F_{\widetilde {R}_{2}}^{T},\qquad \tilde{\Theta}_{1} =\tilde{\Theta}_{2} =- \epsilon_{13}I,\qquad \tilde{\Theta}_{3}=\tilde{ \Theta}_{4}=-\epsilon_{14}I,\qquad \tilde{ \Theta}_{5}=\tilde{\Theta}_{6}=-\epsilon_{15}I, \\& \tilde{\Theta}_{7}=\tilde{\Theta}_{8}=- \epsilon_{16}I,\qquad \tilde{\vartheta}_{2}^{*} = D_{j}^{T}N_{D_{j}}^{T}F_{\widetilde {H}_{j}}^{T},\qquad \tilde{\Theta}_{9} =\tilde{\Theta}_{10} =- \epsilon_{17}I, \\& \tilde{\Theta}_{11}=\tilde{ \Theta}_{12}=-\epsilon_{18}I,\qquad \tilde{ \Theta}_{13}=\tilde{\Theta}_{14}=-\epsilon_{19}I, \qquad \tilde{\Theta}_{15}=\tilde{\Theta}_{16}=- \epsilon_{20}I, \\& \tilde{\Phi}_{1} =\tilde{\Phi}_{2} =-\tilde{ \epsilon}_{13}I,\qquad \tilde{\Phi}_{3}=\tilde{ \Phi}_{4}=-\tilde{\epsilon}_{14}I,\qquad \tilde{ \Phi}_{5}=\tilde{\Phi}_{6}=-\tilde{\epsilon}_{15}I, \\& \tilde{\Phi}_{7}=\tilde{\Phi}_{8}=-\tilde{ \epsilon}_{16}I,\qquad \tilde{\Gamma}_{8}^{*} = \tilde{\epsilon}_{20}F_{\widetilde {H}_{j}}^{T}MN_{D_{j}}^{T},\qquad \tilde{\Phi}_{9} =\tilde{\Phi}_{10} =-\tilde{ \epsilon}_{17}I, \\& \tilde{\Phi}_{11}=\tilde{ \Phi}_{12}=-\tilde{\epsilon }_{18}I,\qquad \tilde{ \Phi}_{13}=\tilde{\Phi}_{14}=-\tilde {\epsilon}_{19}I, \qquad \tilde{\Phi}_{15}=\tilde{\Phi }_{16}=-\tilde{ \epsilon}_{20}I, \qquad \tilde{\Gamma}_{2}^{*} = N_{D_{j}}^{T}\widetilde{H}_{j}^{T}, \\& \vartheta_{1}^{*} = C_{i}^{T}F_{H_{i}}C_{i}+M^{T}N_{C_{i}}^{T}F_{H_{i}}MN_{C_{i}}, \qquad \tilde{\vartheta}_{1}^{*} = D_{j}^{T}F_{\widetilde {H}_{j}}D_{j}+M^{T}N_{D_{j}}^{T}F_{\widetilde{H}_{j}}MN_{D_{j}}, \\& \vartheta_{2}^{*} = C_{i}^{T}N_{C_{i}}^{T}F_{H_{i}}^{T},\qquad \Gamma_{1}^{*} = -C_{i}^{T}F_{H_{i}}^{T}, \qquad \Gamma_{2}^{*} = N_{C_{i}}^{T}H_{i}^{T}, \qquad \Gamma_{3}^{*} = N_{C_{i}}^{T}F_{H_{i}}, \\& \Gamma_{4}^{*} = \epsilon_{15}M \check{E}, \qquad \Gamma_{5}^{*} = N_{C_{i}}H_{i}^{T}, \qquad \tilde{\Gamma}_{5}^{*} = N_{D_{j}} \widetilde{H}_{j}^{T},\qquad \Gamma_{6}^{*} = \epsilon_{17}C_{i}M^{T}, \\& \Gamma_{7}^{*} = \epsilon_{18}M^{T} \check{E}, \qquad \Gamma_{8}^{*} = \epsilon _{20}F_{H_{i}}^{T}MN_{C_{i}}^{T}, \qquad \tilde{\Gamma}_{6}^{*} = \tilde{ \epsilon}_{17}D_{j}M^{T}, \qquad \tilde{ \Gamma}_{7}^{*} = \tilde{\epsilon}_{18}M^{T} \check{E}, \\& \tilde{\Gamma}_{1}^{*} = -D_{j}^{T}F_{\widetilde{H}_{j}}^{T}, \\& \Omega _{99}^{*} = \sum_{l=1}^{N} \tilde{\gamma}_{jl}D_{j}^{T}\widetilde {H}_{l} D_{j}+\sum_{l=1}^{N} \tilde{\gamma }_{jl}M^{T}N_{D_{j}}^{T} \widetilde{H}_{j}MN_{D_{j}}-\widetilde{\eta }_{j}D_{j}^{T}\widetilde{H}_{j}D_{j}+M^{T}N_{D_{j}}^{T} \widetilde {H}_{j}MN_{D_{j}}, \\& \Xi_{29}^{*} = C_{i}^{T}H_{i}C_{i}- \sum_{l=1}^{N}\gamma _{il}C_{i}H_{l}+M^{T}N_{C_{i}}^{T}H_{i}MN_{C_{i}}- \eta _{i}C_{i}^{T}H_{i}, \\& \Omega_{69}^{*} = -D_{j}^{T} \tilde {H}_{j}V_{1j}-M^{T}N_{D_{j}}^{T} \widetilde{H}_{j}MN_{V_{1j}}, \qquad \Xi_{69}^{*} = -C_{i}^{T}H_{i}W_{1i}-M^{T}N_{C_{i}}^{T}H_{i}MN_{W_{1i}}, \\& \Omega _{29}^{*} = D_{j}^{T} \widetilde{H}_{j}D_{j}-\sum_{l=1}^{N} \tilde {\gamma}_{jl} D_{j} \widetilde{H}_{l} +M^{T}N_{D_{j}}^{T}\widetilde {H}_{j}MN_{D_{j}}- \widetilde{\eta}_{j}D_{j}^{T}\widetilde{H}_{j}, \\& \Xi_{99}^{*} = \sum_{l=1}^{N} \gamma_{il}C_{i}^{T}H_{l}C_{i}+ \sum_{l=1}^{N}\gamma_{il}M^{T}N_{C_{i}}^{T}H_{i}MN_{C_{i}}+ \eta _{i}C_{i}^{T}H_{i}C_{i}+M^{T}N_{C_{i}}^{T}H_{i}MN_{C_{i}}, \\& \tilde {\Gamma}_{3}^{*} = N_{D_{j}}^{T}F_{\widetilde{H}_{j}}, \\& \Xi_{79}^{*} = -C_{i}^{T}H_{i}W_{2i}-M^{T}N_{C_{i}}^{T}H_{i}MN_{W_{2i}}, \qquad \Omega _{79}^{*} = -D_{j}^{T} \widetilde {H}_{j}V_{2j}-M^{T}N_{D_{j}}^{T} \widetilde{H}_{j}MN_{V_{2j}}, \\& \tilde{\Gamma}_{4}^{*} = \tilde{\epsilon}_{15}M \check{E},\qquad \Gamma_{15} = \frac{-1}{\sigma_{1}}F_{N_{5}}^{T}, \qquad \tilde {\Gamma}_{15} = \frac{-1}{\sigma_{2}}F_{N_{6}}^{T}, \\& \alpha ^{*} = -(1-\tau_{1})e^{-\eta_{i}\bar{\tau}_{1}}, \qquad \beta^{*} = -(1-\tau_{2})e^{-\widetilde{\eta}_{j}\bar{\tau}_{2}}. \end{aligned}$$

*The remaining values of* $\Xi_{1}^{*}$, $\Delta_{1}^{*}$ *are the same as in Theorem *2.1, *and* ∗ *means the symmetric terms*.

### Proof

The matrices $C_{i}$, $H_{i}$, $D_{j}$, $\widetilde{H}_{j}$, $R_{2}$, $\widetilde{R}_{2}$, *S*, *S̃*, *T*, *T̃*, $N_{1}$, $N_{2}$, $N_{3}$, $N_{4}$, $N_{5}$, and $N_{6}$ in the Lyapunov–Krasovskii functional of Theorem 2.1 are replaced by $C_{i}+\Delta C_{i}(t)$, $H_{i}+\Delta H_{i}$, $D_{j}+\Delta D_{j}(t)$, $\widetilde{H}_{j}+\Delta\widetilde{H}_{j}$, $R_{2}+\Delta R_{2}$, $\widetilde{R}_{2}+\Delta\widetilde{R}_{2}$, $S+\Delta S$, $\widetilde{S}+\Delta\widetilde{S}$, $T+\Delta T$, $\widetilde {T}+\Delta\widetilde{T}$, $N_{1}+\Delta N_{1}$, $N_{2}+\Delta N_{2}$, $N_{3}+\Delta N_{3}$, $N_{4}+\Delta N_{4}$, $N_{5}+\Delta N_{5}$, and $N_{6}+\Delta N_{6}$, respectively.

Hence, by applying the same procedure of Theorem 2.1 and using Assumption 5, Lemmas 1.8, 1.9, 1.10 and 1.11 and putting $\eta= \max_{i,j\in\mathfrak {M}}\{ \max_{i\in\mathfrak{M}}\eta_{i}, \max_{j\in\mathfrak {M}}\widetilde{\eta}_{j}\}$, we have from (28) and Definition 2 (weak infinitesimal operator $\mathcal{L}V$) that

$$ \mathcal{L}V \leq e^{\eta t} \bigl\{ \Psi^{T}(t) \Omega^{*} \Psi (t)+ \Phi^{T}(t)\Pi\Phi(t) \bigr\} , $$

where $\Psi(t)$ and $\Phi(t)$ are given in Theorem 2.1. The remaining proof of this theorem is similar to the procedure of Theorem 2.1, and we get that the uncertain neural network (28) is global robust exponentially stable in the mean square sense. □

## Numerical examples

In this section, we provide two numerical examples with their simulations to demonstrate the effectiveness of our results.

### Example 4.1

Consider the second order stochastic impulsive BAM neural networks (4) with $u(t) = (u_{1}(t),u_{2}(t))^{T}$, $v(t) = (v_{1}(t),v_{2}(t))^{T}$; $\bar{\omega}(t)$, $\bar{\tilde{\omega }}(t)$ are second order Brownian motions and $r(t)$, $\tilde{r}(t)$ denote right-continuous Markovian chains taking values in $\mathfrak {M}=\{1,2\}$ with generator

$$ \Gamma= \begin{bmatrix} 0.2& 0.1\\ 0.4& 0.3 \end{bmatrix} , \qquad \tilde{\Gamma} = \begin{bmatrix} 0.5& 0.2\\ 0.4& 0.3 \end{bmatrix} . $$

The associated parameters of neural networks (4) take the values as follows:

$$\begin{aligned}& C_{1}= \begin{bmatrix} 1& 0\\ 0& 3 \end{bmatrix} ,\qquad C_{2}= \begin{bmatrix} 2& 0\\ 0& 1 \end{bmatrix} ,\qquad D_{1}= \begin{bmatrix} 2& 0\\ 0& 3 \end{bmatrix} ,\qquad D_{2}= \begin{bmatrix} 5& 0\\ 0& 2 \end{bmatrix} , \\& W_{01}= \begin{bmatrix} 0.02& 0.01\\ -0.02& 0.01 \end{bmatrix} ,\qquad W_{02}= \begin{bmatrix} 0.02& 0.01\\ 0.02& 0.01 \end{bmatrix} ,\qquad W_{11}= \begin{bmatrix} 0.03& -0.02\\ 0.03& 0.02 \end{bmatrix} , \\& W_{12}= \begin{bmatrix} 0.02& 0.02\\ -0.01& 0.01 \end{bmatrix} ,\qquad W_{21}= \begin{bmatrix} 0.03& 0.04\\ 0.03& 0.02 \end{bmatrix} ,\qquad W_{22}= \begin{bmatrix} 0.02& -0.02\\ 0.03& -0.01 \end{bmatrix} , \\& V_{01}= \begin{bmatrix} 0.02& 0.02\\ 0.01& 0.03 \end{bmatrix} ,\qquad V_{02}= \begin{bmatrix} 0.02& 0.01\\ 0.01& 0.02 \end{bmatrix} ,\qquad V_{11}= \begin{bmatrix} 0.01& -0.02\\ 0.02& 0.01 \end{bmatrix} , \\& V_{12}= \begin{bmatrix} 0.01& 0.02\\ 0.02& -0.03 \end{bmatrix} ,\qquad V_{21}= \begin{bmatrix} 0.02& 0.01\\ 0.02& 0.01 \end{bmatrix} ,\qquad V_{22}= \begin{bmatrix} 0.02& 0.02\\ 0.03& 0.02 \end{bmatrix} , \\& R_{2}= \begin{bmatrix} 0.02& 0\\ 0& 0.03 \end{bmatrix} ,\qquad \tilde{R}_{2}= \begin{bmatrix} 0.05& 0\\ 0& 0.02 \end{bmatrix} ,\qquad R_{11}= \begin{bmatrix} 0.06& 0\\ 0& 0.04 \end{bmatrix} , \\& R_{12}= \begin{bmatrix} 0.03& 0\\ 0& 0.02 \end{bmatrix} ,\qquad R_{21}= \begin{bmatrix} 0.02& 0\\ 0& 0.03 \end{bmatrix} ,\qquad R_{22}= \begin{bmatrix} 0.05& 0\\ 0& 0.02 \end{bmatrix} , \\& R_{31}= \begin{bmatrix} 0.03& 0\\ 0& 0.08 \end{bmatrix} ,\qquad R_{32}= \begin{bmatrix} 0.07& 0\\ 0& 0.04 \end{bmatrix} ,\qquad \widetilde{R}_{11}= \begin{bmatrix} 0.2341& 0\\ 0& 0.3421 \end{bmatrix} , \\& \widetilde{R}_{12}= \begin{bmatrix} 0.2451& 0\\ 0& 0.0251 \end{bmatrix} ,\qquad \widetilde{R}_{21}= \begin{bmatrix} 0.1802& 0\\ 0& 0.0102 \end{bmatrix} , \\& \widetilde{R}_{22}= \begin{bmatrix} 0.1212& 0\\ 0& 0.0140 \end{bmatrix} ,\qquad \widetilde{R}_{31}= \begin{bmatrix} 0.02& 0\\ 0& 0.05 \end{bmatrix} ,\qquad \widetilde{R}_{32}= \begin{bmatrix} 0.03& 0\\ 0& 0.04 \end{bmatrix} , \\& \bar{M}_{k}= \begin{bmatrix} 0.04& 0\\ 0& 0.05 \end{bmatrix} ,\qquad \bar{N}_{k}= \begin{bmatrix} 0.05& 0\\ 0& 0.03 \end{bmatrix} . \end{aligned}$$

Taking

$$\begin{aligned}& \bar{\rho}_{1}\bigl(u(t-\nu_{1}),v(t),v\bigl(t- \tau_{1}(t)\bigr),t,1\bigr) \\& \quad= \begin{bmatrix} 0.04*u_{1}(t-\nu_{1})+0.04*v_{1}(t)+0.03*v_{1}(t-\bar{\tau}_{1})& 0\\ 0& 0.05*u_{2}(t-\nu_{1})+0.02*v_{2}(t)+0.03*v_{2}(t-\bar{\tau}_{1}) \end{bmatrix} , \\& \bar{\rho}_{1}\bigl(u(t-\nu_{1}),v(t),v\bigl(t- \tau_{1}(t)\bigr),t,2\bigr) \\& \quad= \begin{bmatrix} 0.04*u_{1}(t-\nu_{1})+0.03*v_{1}(t)+0.05*v_{1}(t-\bar{\tau}_{1})& 0\\ 0& 0.02*u_{2}(t-\nu_{1})+0.04*v_{2}(t)+0.02*v_{2}(t-\bar{\tau}_{1}) \end{bmatrix} , \\& \bar{\rho}_{2}\bigl(v(t-\nu_{2}),u(t),u\bigl(t- \tau_{2}(t)\bigr),t,1\bigr) \\& \quad= \begin{bmatrix} 0.02*v_{1}(t-\nu_{2})+0.02*u_{1}(t)+0.02*u_{1}(t-\bar{\tau}_{2})& 0\\ 0& 0.03*v_{2}(t-\nu_{2})+0.02*u_{2}(t)+0.05*u_{2}(t-\bar{\tau}_{2}) \end{bmatrix} , \\& \bar{\rho}_{2}\bigl(v(t-\nu_{2}),u(t),u\bigl(t- \tau_{2}(t)\bigr),t,2\bigr) \\& \quad= \begin{bmatrix} 0.01*v_{1}(t-\nu_{2})+0.03*u_{1}(t)+0.03*u_{1}(t-\bar{\tau}_{2})& 0\\ 0& 0.04*v_{2}(t-\nu_{2})+0.03*u_{2}(t)+0.02*u_{2}(t-\bar{\tau}_{2}) \end{bmatrix} , \\& \nu_{1} = \nu_{2} = 1 , \qquad \bar{\tau}_{1} = \bar{\tau}_{2} = 7.46 , \qquad \sigma_{1} = 0.6 ,\qquad \sigma_{2} = 0.8 . \end{aligned}$$

The following activation functions play in neural network system (4):

$$\begin{aligned}& \bar{f}(v) = \sinh(v),\qquad \bar{\tilde{f}}(u) = \sinh (u),\qquad \bar{g}(v) = v, \\& \bar{\tilde{g}}(u) = u, \qquad\bar {h}(v) = \sin(v), \qquad \bar{\tilde{h}}(u) = \sin(u). \end{aligned}$$

It is easy to obtain that, for any $a, b \in\mathbb{R}$ with $a < b$, there exists a scalar $c \in(a,b)$ such that

$$ \frac{f_{i}(b)-f_{i}(a)}{b-a} = \frac{\sinh(b)-\sinh(a)}{b-a} =\cosh(c) \geq1. $$

Therefore $f_{i}(\cdot)$ and $\bar{\tilde{f}}_{j}(\cdot)$, $i,j = 1,2$, are 1-inverse Holder functions. In addition, for any $a, b \in\mathbb {R}$, it is easy to check that

$$\begin{aligned} \bigl\vert h_{i}(b)-h_{i}(a) \bigr\vert =& \bigl\vert \sin(b)-\sin(a) \bigr\vert \leq \bigl\vert g_{i}(b)-g_{i}(a) \bigr\vert = \vert b-a \vert \\ \leq& \bigl\vert f_{i}(b)-f_{i}(a) \bigr\vert = \bigl\vert \sinh(b)-\sinh(a) \bigr\vert . \end{aligned}$$

By a similar way, we get the same inequalities for $\bar{\tilde {g}}_{j}(\cdot)$ and also $\bar{\tilde{h}}_{j}(\cdot)$ ($j=1,2$). That means the activation functions $\bar{f}_{i}(\cdot)$, $\bar {\tilde{f}}_{j}(\cdot)$, $\bar{g}_{i}(\cdot)$, $\bar{\tilde {g}}_{j}(\cdot)$, $\bar{h}_{i}(\cdot)$, $\bar{\tilde{h}}_{j}(\cdot )$ ($i, j=1,2$) satisfy Assumptions 1 and 2.

Then, by Theorem 2.1, solving the LMIs using the Matlab LMI control toolbox, one can obtain the following feasible solutions:

$$\begin{aligned}& S=10^{-4} \times \begin{bmatrix} 0.2968& -0.0000\\ -0.0000& 0.2957 \end{bmatrix} ,\qquad T = 10^{-4} \times \begin{bmatrix} 0.8435& -0.0000\\ -0.0000& 0.8461 \end{bmatrix} , \\& N_{1} = \begin{bmatrix} 0.4948& -0.0001\\ -0.0001& 0.4948 \end{bmatrix} , \qquad N_{3} = \begin{bmatrix} 0.7465& 0.0147\\ 0.0147& 0.7263 \end{bmatrix} , \\& N_{4} = \begin{bmatrix} 0.8333& -0.0046\\ -0.0046& 0.8248 \end{bmatrix} ,\qquad N_{5} = \begin{bmatrix} 0.4424& 0.0001\\ 0.0001& 0.4423 \end{bmatrix} , \\& \widetilde{S} = \begin{bmatrix} 0.0373& 0.0000\\ 0.0000& 0.0373 \end{bmatrix} ,\qquad \widetilde{T} = 10^{-3} \times \begin{bmatrix} 0.2156& -0.0001\\ -0.0001& 0.2155 \end{bmatrix} , \\& H_{1} = \begin{bmatrix} 0.3533& -0.0069\\ -0.0069& 0.0936 \end{bmatrix} , \qquad \widetilde{H}_{1} = \begin{bmatrix} 0.0728& 0\\ 0& 0.0076 \end{bmatrix} , \\& \widetilde{H}_{2} = \begin{bmatrix} 0.0355& -0.0030\\ -0.0030& 0.0350 \end{bmatrix} ,\qquad P= 10^{-4} \times \begin{bmatrix} 0.4368& 0\\ 0& 0.4487 \end{bmatrix} , \\& N_{2} = \begin{bmatrix} 0.4979& 0.0215\\ 0.0215& 0.9157 \end{bmatrix} ,\qquad N_{6} = \begin{bmatrix} 0.4841& 0.0000\\ 0.0000& 0.4840 \end{bmatrix} , \\& H_{2} = \begin{bmatrix} 0.0016& -0.0000\\ -0.0000& 0.0017 \end{bmatrix} ,\qquad P_{1}= 10^{-4} \times \begin{bmatrix} 0.4947& 0\\ 0& 0.4947 \end{bmatrix} , \\& Q_{1}= 10^{-3} \times \begin{bmatrix} 0.4947& 0\\ 0& 0.4947 \end{bmatrix} ,\qquad Q= 10^{-3} \times \begin{bmatrix} 0.5549& 0\\ 0& 0.4465 \end{bmatrix} , \\& \lambda_{1} = 0.6805,\qquad \lambda_{2} = 0.0023, \qquad \mu_{1} = 2.0060, \qquad\mu_{2} = 0.8234. \end{aligned}$$

**Figure 1:**
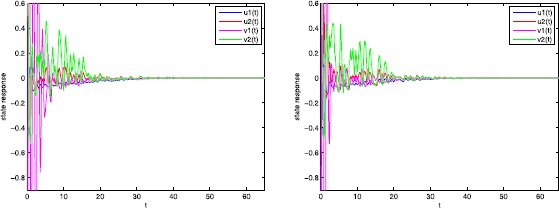
The state response $u_{1}(t)$, $u_{2}(t)$, $v_{1}(t)$, $v_{2}(t)$ of (1) with stochastic disturbances and without stochastic disturbances

**Figure 2:**
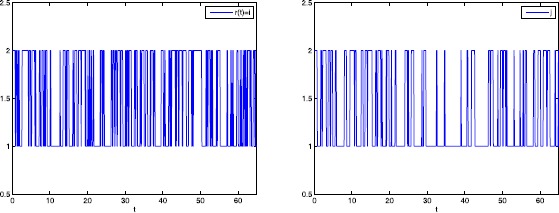
The state responses $r(t)$ and $\tilde{r}(t)$ denote Markovian jump in system (4)

Figure 1 narrates the time response of state variables $u_{1}(t)$, $u_{2}(t)$, $v_{1}(t)$, $v_{2}(t)$ with and without stochastic noises, and Fig. 2 depicts the time response of Markovian jumps $r(t)=i$, $\tilde{r}(t)=j$. By solving LMIs (5)–(10), we get the feasible solutions. The obtained discrete time delay upper bounds of $\bar{\tau}_{1}$ and $\bar{\tau}_{2}$ for neural networks (4), which are given in Table 1, are very maximum. This shows that the contributions of this research work is more effective and less conservative than some existing results. Therefore, by Theorem 2.1, we can conclude that neural networks (4) are globally exponentially stable in the mean square for the maximum allowable upper bounds $\bar{\tau}_{1} = \bar{\tau}_{2} = 7.46$.

**Table 1:** Maximum allowable upper bounds of discrete time delays

Methods	$\bar{\tau}_{1} = \bar{\tau}_{2} > 0$	System status
In Ref. [41]	0.5784	feasible
In Ref. [42]	2.1	feasible
In Ref. [43]	4.822	feasible
In Ref. [44]	5	feasible
In Ref. [34]	5.912	feasible
In Ref. [45]	6.884	feasible
Theorem 2.1	7.46	feasible

### Example 4.2

Consider the second order uncertain stochastic impulsive BAM neural networks (28) with $u(t) = (u_{1}(t), u_{2}(t))^{T}$, $v(t) = (v_{1}(t),v_{2}(t))^{T}$; $\bar{\omega}(t)$, $\bar{\tilde{\omega }}(t)$ are second order Brownian motions and $r(t)$, $\tilde{r}(t)$ denote right-continuous Markovian chains taking values in $\mathfrak {M}=\{1,2\}$ with generator

$$\begin{aligned} \Gamma= \widetilde{\Gamma} = \begin{bmatrix} -3& 3\\ 4& -2 \end{bmatrix} . \end{aligned}$$

The associated parameters of neural networks (28) are as follows:

$$\begin{aligned}& C_{1}= \begin{bmatrix} 1& 0\\ 0& 3 \end{bmatrix} ,\qquad C_{2}= \begin{bmatrix} 2& 0\\ 0& 1 \end{bmatrix} ,\qquad D_{1}= \begin{bmatrix} 2& 0\\ 0& 1 \end{bmatrix} ,\qquad D_{2}= \begin{bmatrix} 2& 0\\ 0& 2 \end{bmatrix} , \\& W_{01}= \begin{bmatrix} 0.02& 0.01\\ -0.02& 0.01 \end{bmatrix} , \qquad W_{02}= \begin{bmatrix} 0.02& 0.01\\ 0.02& 0.01 \end{bmatrix} ,\qquad W_{11}= \begin{bmatrix} 0.03& -0.02\\ 0.03& 0.02 \end{bmatrix} , \\& W_{12}= \begin{bmatrix} 0.02& 0.02\\ -0.01& 0.01 \end{bmatrix} ,\qquad W_{21}= \begin{bmatrix} 0.05& 0.01\\ 0.03& 0.02 \end{bmatrix} , \qquad W_{22}= \begin{bmatrix} 0.02& -0.01\\ 0.03& -0.01 \end{bmatrix} , \\& V_{01}= \begin{bmatrix} 0.02& 0.02\\ 0.01& 0.03 \end{bmatrix} ,\qquad V_{02}= \begin{bmatrix} 0.02& 0.01\\ 0.01& 0.02 \end{bmatrix} ,\qquad V_{11}= \begin{bmatrix} 0.01& -0.02\\ 0.02& 0.01 \end{bmatrix} , \\& V_{12}= \begin{bmatrix} 0.01& 0.02\\ 0.02& -0.03 \end{bmatrix} ,\qquad V_{21}= \begin{bmatrix} 0.02& 0.01\\ 0.02& 0.01 \end{bmatrix} ,\qquad V_{22}= \begin{bmatrix} 0.02& 0.02\\ 0.03& 0.02 \end{bmatrix} , \\& M= \begin{bmatrix} 0.5& 0.6\\ 0.2& 0.5 \end{bmatrix} ,\qquad N_{C_{1}}= \begin{bmatrix} 0.1& 0\\ 0& 0.3 \end{bmatrix} ,\qquad N_{C_{2}}= \begin{bmatrix} 0.2& 0\\ 0& 0.2 \end{bmatrix} , \\& N_{D_{1}}= \begin{bmatrix} 0.1& 0\\ 0& 0.3 \end{bmatrix} ,\qquad N_{D_{2}}= \begin{bmatrix} 0.2& 0\\ 0& 0.2 \end{bmatrix} , \qquad N_{W_{01}}= \begin{bmatrix} 0.05& 0.06\\ 0.02& 0.02 \end{bmatrix} , \\& N_{W_{02}}= \begin{bmatrix} 0.02& 0.06\\ 0.02& 0.02 \end{bmatrix} ,\qquad N_{W_{11}}= \begin{bmatrix} 0.03& 0.04\\ 0.02& 0.01 \end{bmatrix} ,\qquad N_{W_{12}}= \begin{bmatrix} 0.01& 0.03\\ 0.03& 0.01 \end{bmatrix} , \\& N_{W_{21}}= \begin{bmatrix} 0.04& 0.03\\ 0.03& 0.02 \end{bmatrix} ,\qquad N_{W_{22}}= \begin{bmatrix} 0.02& 0.03\\ 0.02& 0.01 \end{bmatrix} ,\qquad N_{V_{01}}= \begin{bmatrix} 0.03& 0.06\\ 0.02& 0.02 \end{bmatrix} , \\& N_{V_{02}}= \begin{bmatrix} 0.02& 0.04\\ 0.01& 0.03 \end{bmatrix} , \qquad N_{V_{11}}= \begin{bmatrix} 0.02& 0.04\\ 0.02& 0.03 \end{bmatrix} ,\qquad N_{V_{12}}= \begin{bmatrix} 0.06& 0.03\\ 0.01& 0.04 \end{bmatrix} , \\& N_{V_{21}}= \begin{bmatrix} 0.05& 0.05\\ 0.03& 0.01 \end{bmatrix} ,\qquad N_{V_{22}}= \begin{bmatrix} 0.03& 0.03\\ 0.02& 0.03 \end{bmatrix} . \end{aligned}$$

Taking

$$\begin{aligned}& \bar{\rho}_{1}\bigl(u(t-\nu_{1}),v(t),v\bigl(t- \tau_{1}(t)\bigr),t,1\bigr) \\& \quad = \begin{bmatrix} 0.3*u_{1}(t-\nu_{1})+0.4*v_{1}(t)+0.4*v_{1}(t-\bar{\tau}_{1})& 0\\ 0& 0.3*u_{2}(t-\nu_{1})+0.2*v_{2}(t)+0.3*v_{2}(t-\bar{\tau}_{1}) \end{bmatrix} , \\& \bar{\rho}_{1}\bigl(u(t-\nu_{1}),v(t),v\bigl(t- \tau_{1}(t)\bigr),t,2\bigr) \\& \quad= \begin{bmatrix} 0.4*u_{1}(t-\nu_{1})+0.3*v_{1}(t)+0.4*v_{1}(t-\bar{\tau}_{1})& 0\\ 0& 0.2*u_{2}(t-\nu_{1})+0.5*v_{2}(t)+0.2*v_{2}(t-\bar{\tau}_{1}) \end{bmatrix} , \\& \bar{\rho}_{2}\bigl(v(t-\nu_{2}),u(t),u\bigl(t- \tau_{2}(t)\bigr),t,1\bigr) \\& \quad= \begin{bmatrix} 0.3*v_{1}(t-\nu_{2})+0.2*u_{1}(t)+0.2*u_{1}(t-\bar{\tau}_{2})& 0\\ 0& 0.4*v_{2}(t-\nu_{2})+0.5*u_{2}(t)+0.3*u_{2}(t-\bar{\tau}_{2}) \end{bmatrix} , \\& \bar{\rho}_{2}\bigl(v(t-\nu_{2}),u(t),u\bigl(t- \tau_{2}(t)\bigr),t,2\bigr) \\& \quad= \begin{bmatrix} 0.2*v_{1}(t-\nu_{2})+0.3*u_{1}(t)+0.4*u_{1}(t-\bar{\tau}_{2})& 0\\ 0& 0.2*v_{2}(t-\nu_{2})+0.3*u_{2}(t)+0.2*u_{2}(t-\bar{\tau}_{2}) \end{bmatrix} , \end{aligned}$$

$\nu_{1} = \nu_{2} = 1$, $\bar{\tau}_{1} = \bar{\tau}_{2} = 0.4$, $\sigma_{1} = \sigma_{2} = 0.3$. The following activation functions play in neural network system (28):

$$\begin{aligned}& \bar{f}(v) = \sinh(v), \qquad \bar{\tilde{f}}(u) = \sinh (u),\qquad \bar{g}(v) = v, \\& \bar{\tilde{g}}(u) = u, \qquad \bar {h}(v) = \sin(v), \qquad \bar{\tilde{h}}(u) = \sin(u). \end{aligned}$$

Therefore, by Theorem 3.2 in this paper, the uncertain delayed stochastic impulsive BAM neural networks (28) under consideration are global robust exponentially stable in the mean square.

## Conclusions

In this paper, we have treated the problem of global exponential stability analysis for the leakage delay terms. By employing the Lyapunov stability theory and the LMI framework, we have attained a new sufficient condition to justify the global exponential stability of stochastic impulsive uncertain BAMNNs with two kinds of time-varying delays and leakage delays. The advantage of this paper is that different types of uncertain parameters were introduced into the Lyapunov–Krasovskii functionals, and the exponential stability behavior was studied. Additionally, two numerical examples have been provided to reveal the usefulness of our obtained deterministic and uncertain results. To the best of our knowledge, there are no results on the exponential stability analysis of inertial-type BAM neural networks with both time-varying delays by using Wirtinger based inequality, which might be our future research work.
